# Electrochemical activation of C–H by electron-deficient W_2_C nanocrystals for simultaneous alkoxylation and hydrogen evolution

**DOI:** 10.1038/s41467-021-24203-8

**Published:** 2021-06-23

**Authors:** Xiu Lin, Shi-Nan Zhang, Dong Xu, Jun-Jun Zhang, Yun-Xiao Lin, Guang-Yao Zhai, Hui Su, Zhong-Hua Xue, Xi Liu, Markus Antonietti, Jie-Sheng Chen, Xin-Hao Li

**Affiliations:** 1 School of Chemistry and Chemical Engineering, Shanghai Jiao Tong University, Shanghai, China; 2 Department of Colloid Chemistry, Max-Planck Institute of Colloids and Interfaces, Wissenschaftspark Golm, Potsdam, Germany

**Keywords:** Organic chemistry, Electrocatalysis, Nanoscale materials

## Abstract

The activation of C–H bonds is a central challenge in organic chemistry and usually a key step for the retro-synthesis of functional natural products due to the high chemical stability of C–H bonds. Electrochemical methods are a powerful alternative for C–H activation, but this approach usually requires high overpotential and homogeneous mediators. Here, we design electron-deficient W_2_C nanocrystal-based electrodes to boost the heterogeneous activation of C–H bonds under mild conditions via an additive-free, purely heterogeneous electrocatalytic strategy. The electron density of W_2_C nanocrystals is tuned by constructing Schottky heterojunctions with nitrogen-doped carbon support to facilitate the preadsorption and activation of benzylic C–H bonds of ethylbenzene on the W_2_C surface, enabling a high turnover frequency (18.8 h^−1^) at a comparably low work potential (2 V versus SCE). The pronounced electron deficiency of the W_2_C nanocatalysts substantially facilitates the direct deprotonation process to ensure electrode durability without self-oxidation. The efficient oxidation process also boosts the balancing hydrogen production from as-formed protons on the cathode by a factor of 10 compared to an inert reference electrode. The whole process meets the requirements of atomic economy and electric energy utilization in terms of sustainable chemical synthesis.

## Abstract (web summary)

Designing electrode materials for mild and additive-free activation of C–H bonds is of great challenge. The authors report the application of electron-deficient W_2_C nanocrystal electrodes to boost the dissociation of C-H bonds toward the efficient alkoxylation and hydrogen evolution reactions.

## Introduction

Direct activation of C–H bonds via selective oxidation of hydrocarbons is of great interest for organic hydrocarbons^1,2^. As a typical and important transformation path of C–H bonds, selective dehydrogenation of C–H bonds have been widely used for the production of high value-added compounds such as alcohols, and ketones, and ethers^3–5^. However, the relative stability of C(*sp*^*3*^)−H bonds adjacent to aromatic rings make C–H activation quite challenging, and either extreme and rather toxic oxidants as chromium or selenium compounds or noble-metal-catalysts (based on rhodium or palladium) at high temperatures have to be applied to obtain acceptable conversions^6–8^. Moreover, the as-formed side product water from the cleavage of C–H bonds via oxydehydrogenation is free of value. As a result, sustainable strategies are highly desirable to further decrease the economic and environmental footprints of C−H activation processes.

Electrochemical transformation is recognized as an environmentally friendly method for the production of various functional molecules driven by electricity under mild conditions^9–11^. Pioneering works of electrochemical synthesis using homogeneous catalysts have demonstrated the advantages of this technique for C–H activation^11–13^, which includes selective oxidation^14^, amination^15^, epoxidation^16^, and dehydrogenative coupling reactions^17^. Most of these reactions have a high atom economy and excellent compatibility with flow reactors for continuous synthesis^18,19^. The current strategies to boost the transformation of specific substrates mainly rely on the involvement of functional additives^11,20^ (e.g., organic ligands, bases, and mediators), a high work potential, and/or sacrificial transition metal electrodes^21^, which all will severely limit real-industry applications. Principally, the preparation of cost-effective and active electrode materials is at least as important as the development of a methodology for selective C–H bonds activation^22–29^, and only non-targeted, commercial first-generation electrodes (such as carbon rod, platinum, and reticulated vitreous carbon) are applied as the current collectors in electrochemical organic synthesis at the moment. The significant progress reported using well-designed reaction-specified electrodes in improving the catalytic activity for water splitting, nitrogen reduction reactions, and even carbon dioxide reduction reactions^30–33^ further manifests the huge gap between the design of electrode materials and the requirements of sustainable electrochemical organic synthesis.

Herein, we present the proof-of-concept application of electron-deficient W_2_C nanocrystal-based electrodes for the highly efficient electrochemical activation of C–H bonds, highlighting the key importance of the modified physicochemical properties of electrode materials in boosting additive-free C–H activation reactions. A nanoheterojunction composed of W_2_C nanocrystals and nitrogen-doped carbons has been rationally designed to control the number of electrons flowing from W_2_C nanocrystals to nitrogen-doped carbons by increasing the doping concentration in the carbon supports to enhance the interfacial Schottky effect. The as-formed electron-deficient W_2_C nanocrystal-based electrode acts as a functional anode to simultaneously facilitate the alkoxylation of ethylbenzene with methanol on the anode and the balancing hydrogen evolution reaction on the cathode. Both the experimental and theoretical results indicate the key role of the electron deficiency of the W_2_C nanocrystals in capturing ethylbenzene on the anode to substantially increase the reaction rates of alkoxylation and hydrogen evolution reaction processes simultaneously and ensure the stability of the anode without scarifying the current collector.

## Results

### Preparation and characterization of W_2_C/N_*x*_C

**Fig. 1:**
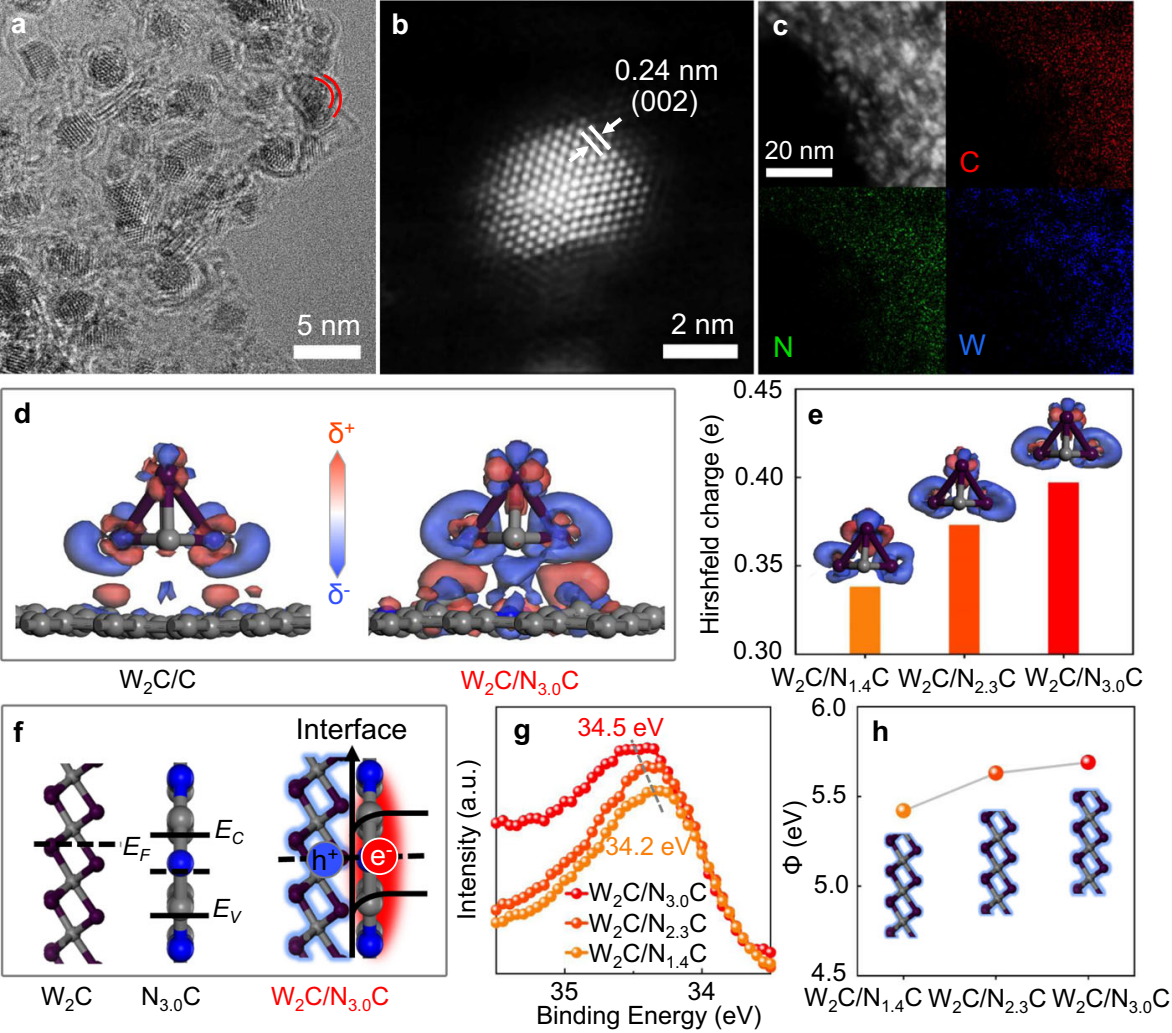
Physical characterizations of the electron-deficient W_2_C nanocrystals on the N_x_C. **a** High-resolution TEM image of W_2_C/N_3.0_C. **b** A HAADF-STEM image of W_2_C/N_3.0_C. **c** Energy-dispersive X-ray elemental mapping of W_2_C/N_3.0_C. **d** Electron density difference stereograms of W_2_C/C and W_2_C/N_3.0_C models (electron-deficient area (δ^−^), blue; electron-rich area (δ^+^), red); color code: C, gray; N, blue; W, purple. The small W_4_C_2_ cluster model represents the W_2_C nanocrystal. **e** The numbers of electrons flowing from W_2_C nanocrystals to various N_*x*_C support models. **f** Schematic diagrams of the rectifying interface of W_2_C and N_3.0_C. **g** W 4*f* XPS spectra of W_2_C/N_*x*_C. **h** Work functions (Φ) of W_2_C/N_*x*_C.

The W_2_C/NC catalysts were prepared via a modified nanoconfinement method (Supplementary Fig. 1) from a mixture of dicyandiamide and ammonium tungstate, followed by N_2_-protected thermal pyrolysis at high temperatures. The nitrogen contents (*x* at.%) of the W_2_C/N_*x*_C samples could be tuned from 3.0 via 2.3 to 1.4 at.% (Supplementary Fig. 2 and Table 1) by elevating the condensation temperatures from 1000 to 1200 °C (for experimental details please see the experimental section). The morphology (Supplementary Fig. 3), surface area (Supplementary Fig. 4), structure (Supplementary Fig. 5), and W content (Supplementary Table 1) of W_2_C/N_*x*_C samples are well maintained, as reflected by their scanning electron microscopy (SEM) images. Transmission electron microscopy (TEM) observations (Fig. 1a–c and Supplementary Figs. 6–8) further reveal the presence of few-layer-graphene-supported W_2_C nanocrystals with a mean size of 2.5 nm (Fig. 1a and Supplementary Fig. 9) and a typical lattice fringe of 0.24 nm (Fig. 1b), which corresponds to the (002) plane of α-W_2_C^34,35^. The formation of W_2_C is doubly confirmed by its X-ray diffraction (XRD) pattern (Supplementary Fig. 10), matching well with that of typical α-W_2_C (JCPDS# 35-776)^34^. Detailed elemental mapping images (Fig. 1c) exhibit nanometer-sized W-rich areas with a homogeneous distribution of N atoms along with the whole carbon support, indicating an integrated structure of W_2_C nanocrystals on the nitrogen-doped carbons.

### Identification of electron-deficient W_2_C nanocrystals

The highly coupled structure of W_2_C/NC dyads makes it possible to form a rectifying interface for modulation of the electron density of W_2_C nanocrystals. The density functional theory (DFT) calculation results (Supplementary Figs. 11, 12) predict electron transfer from W_2_C to nitrogen-doped carbons, resulting in more pronounced electron-deficient regions in W_2_C nanocrystals suggested by the electron density difference stereograms (Fig. 1d) of the same W_2_C model supported on pristine carbons (W_2_C/C). The total number of electrons transferred from the small W_4_C_2_ cluster to the nitrogen-doped carbon support (Fig. 1e) increases from 0.338 to 0.397 as more nitrogen atoms (from 1.4 to 3.0 at.%) are doped into the carbon support models (Supplementary Fig. 12j–l) on the basis of the X-ray photoelectron spectroscopy (XPS) analysis results^36^, while the trend is not affected by the cluster stoichiometry (Supplementary Fig. 13). As depicted in Fig. 1f, the nanoheterojunction of W_2_C and NC has a rectifying contact, with electrons flowing from the W_2_C side with a lower Fermi level (*E*_F_) to the NC side, generating electron-deficient W_2_C due to the interfacial Schottky barrier^37,38^. Indeed, the electron donation from the W_2_C nanocrystals to the nitrogen-rich carbon supports is experimentally confirmed by the gradual shift in W 4*f* XPS peaks to higher energy (Fig. 1g) from 34.2 via 34.4 to 34.5 eV for W_2_C/N_1.4_C, W_2_C/N_2.3_C, and W_2_C/N_3.0_C, respectively, resulting in gradually increased work functions (Fig. 1h and Supplementary Fig. 14) from 5.4 via 5.6 to 5.7 eV. A similar trend for the electron density of W_2_C nanocrystals in W_2_C/N_*x*_C samples is also demonstrated by the most positive W M4,5 peak (Supplementary Fig. 15) of the W_2_C/N_3.0_C materials among all samples^39^. All of the above results indicate the formation of electron-deficient W_2_C nanocrystals and the successful further enhancement of electron deficiencies by increasing the nitrogen contents in the carbon supports.

### Catalytic performance and reaction pathways of catalysts

**Fig. 2:**
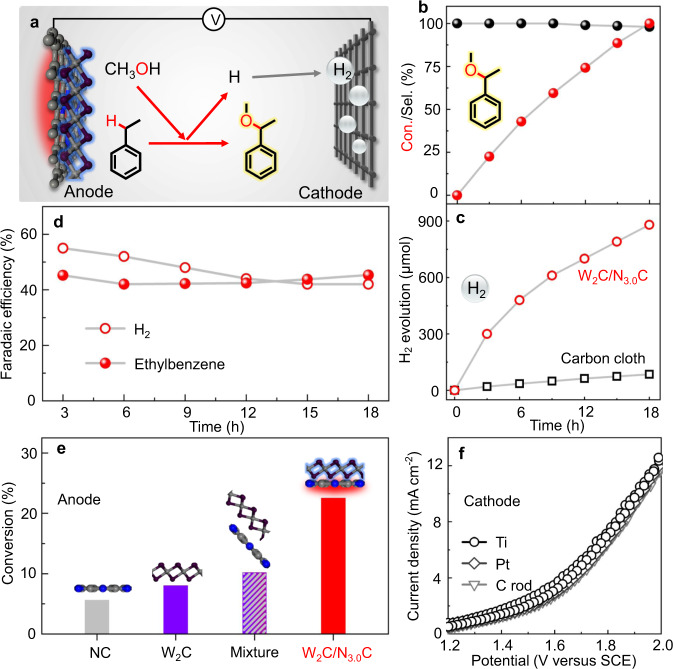
Catalytic performance of the electron-deficient W_2_C nanocrystal-based anode. **a** Schematic illustration of electrolyzer and proposed reaction pathway. **b** The time courses of conversion (red) of ethylbenzene and selectivity (black) to (1-methoxyethyl)benzene on the W_2_C/N_3.0_C anode. **c** The hydrogen production at the cathode when W_2_C/N_3.0_C and CC were used as the anode. **d** The time courses of Faradaic efficiencies for conversion of ethylbenzene on the anode and hydrogen production on the cathode, respectively. **e** Conversions of ethylbenzene on W_2_C/N_3.0_C and control anodes under standard conditions within 3 h. **f** Cyclic voltammetry curves of typical cathodes (Ti mesh, Pt mesh, and C rod) with the same W_2_C/N_3.0_C anode. Standard reaction conditions: ethylbenzene (0.5 mmol), lithium perchlorate (1 mmol), methanol (15 mL), W_2_C/N_3.0_C anode, and Ti mesh cathode at 2.0 V versus SCE at room temperature in a home-made electrolyzer (Supplementary Fig. 16).

Inspired by the success in modifying the electron density of W_2_C nanocrystals, we further evaluated the possible catalytic activity of W_2_C/N_*x*_C catalysts for electrochemical alkoxylation of ethylbenzene with methanol under mild conditions as a model reaction. Considering that the reported methods for alkoxylation of C–H bonds usually require highly active additives/oxidants and/or a high reaction temperature, we initially tested the possibility of additive-free alkoxylation of ethylbenzene with methanol using only a simple electrolyte containing lithium perchlorate and W_2_C/N_*x*_C-based electrodes under ambient conditions (Fig. 2 and Supplementary Fig. 16). No product was detected without applying a working potential for various electrodes in our electrochemical system (Supplementary Fig. 17a–c), illustrating that the methoxylation reaction cannot proceed spontaneously. Widely used commercial electrodes, including reticulated vitreous carbon (RVC), boron-doped diamond (BDD), and lead oxide, exhibit poor activity (Supplementary Fig. 17d) in our additive-free electrocatalytic system. Surprisingly, a complete conversion of ethylbenzene can be achieved on the W_2_C/N_3.0_C electrode with high selectivity to the target product (1-methoxyethyl)benzene (Fig. 2a, b and Supplementary Fig. 18) and a total carbon balance of approximately 95%, confirming the possibility of highly efficient alkoxylation of C–H bonds on a well-designed heterogeneous electrode without scarifying additives. The fact that control electrodes with the same amount of bare NC sample, W_2_C catalyst, or a mechanical mixture of the two components (Fig. 2e) give much lower conversions of ethylbenzene than the W_2_C/N_3.0_C electrode under fixed conditions further indicates a synergistic effect between W_2_C and N_3.0_C components in facilitating the transformation of ethylbenzene.

Unlike the oxidative alkoxylation reaction of C–H bonds by using various oxidants for dehydrogenation to generate water^40^, our heterogeneous electrochemical system could achieve the full use of as-formed protons from the activation of C–H bonds and methanol for subsequent hydrogen evolution reactions, generating hydrogen gas bubbles on the cathode (Supplementary Fig. 19 and Supplementary Movie 1). Moreover, the calculated Faradaic efficiencies (Fig. 2d and Supplementary Fig. 20) are similar for the conversion of ethylbenzene to (1-methoxyethyl)benzene on the W_2_C/N_3.0_C anode (*F*_E_: 42–46%) and hydrogen production on the Ti cathode (*F*_E_: 42–55%), implying a cascade transformation of protons generated from the anode into hydrogen gas on the cathode. Even with an excess amount of methanol in the reactor, only a trace amount of formaldehyde (0.006 mmol) formed during the conversion of 0.5 mmol of ethylbenzene (Supplementary Fig. 21), well explaining the comparable Faradaic efficiencies for the reactions on anode and cathode without the obvious contribution of methanol dehydrogenation to the total *F*_E_ for hydrogen evolution reactions. Remarkably, the electron-deficient W_2_C in the W_2_C/N_3.0_C-based electrode substantially promotes the hydrogen evolution rate on the Ti cathode to 880 μmol (Fig. 2c), which is above 10 times that on the same Ti cathode (85 μmol) when using bare carbon cloth as the anode. The direct release of hydrogen gas on the counter electrode directly confirms the C–H bond activation via single-electron oxidation followed by deprotonation, because as-formed hydrogen atoms from direct hydrogen abstraction could not transfer from W_2_C-based working electrode via the electrolyte solution to the counter electrode for hydrogen gas production. The constant current density of the W_2_C/N_3.0_C anode under fixed conditions with different cathodes (Fig. 2f), including Pt mesh, Ti mesh, and carbon rod, further demonstrates that the activation of ethylbenzene on the W_2_C/N_3.0_C electrode is the rate dominating step for the whole reaction. The reaction pathways are reasonably proposed via deprotonation of ethylbenzene and followed by methanol addition on the basis of the additional quenching experiments with an additional oxidation peak (Supplementary Fig. 22) after the addition of butylated hydroxytoluene (BHT), and no observable signals of any possible radicals were detected, as indicated in Fig. 2a^41,42^.

### Investigation of the mechanism at heterojunction interfaces

**Fig. 3:**
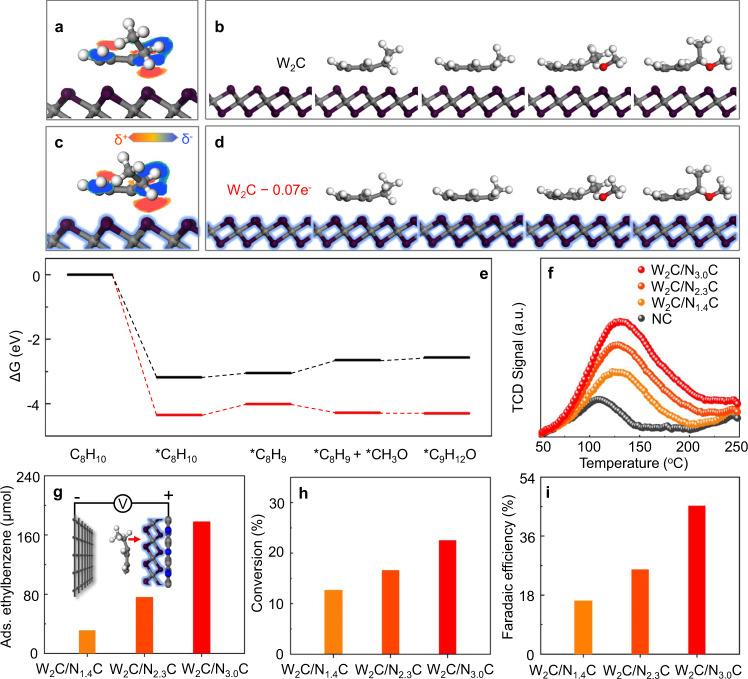
Effect of the electron deficiency of W_2_C nanocrystals on the conversion of ethylbenzene. **a**, **c** Electron density difference stereograms of benzylic C–H bonds of ethylbenzene on the surface of W_2_C model (**a**) and electron-deficient W_2_C model (W_2_C-0.07e^−^) (**b**). The slice is perpendicular to the plane of benzylic C–H bonds, and electron-rich (red) and electron-deficient (blue) areas are presented. color code: C, gray; N, blue; H, white; W, purple. **c**, **d** Calculated absorption configurations of each step of the ethylbenzene activation process on W_2_C (**c**) and W_2_C-0.07e^−^ (**d**). **e** Gibbs free energy diagrams of each step of the ethylbenzene activation process on W_2_C (black and top) and W_2_C-0.07e^−^ (red and bottom). **f** Ethylbenzene-TPD results of the W_2_C/N_*x*_C and NC samples. **g** Adsorption capacities of ethylbenzene molecules on the surface of the W_2_C/N_x_C electrodes with fixed catalyst loadings (0.66 mg/cm^2^) at 2.0 V versus SCE for 10 min. **h** Conversions of ethylbenzene on W_2_C/N_x_C electrodes at 2.0 V versus SCE for 3 h. **i** Faradaic efficiencies for ethylbenzene conversion on W_2_C/N_x_C electrodes at 2.0 V versus SCE for 3 h.

The role of the electron-deficient W_2_C nanocrystals and the interfacial effect of the heterojunction catalysts on the electrochemical alkoxylation of C–H bonds were simulated via theoretical calculations and then validated by experimental evidence (Fig. 3). The optimized geometry (Fig. 3a, c) of ethylbenzene presents the preferred adsorption of benzylic C–H bonds on the W_2_C surface dependent on the electron-deficiency of W_2_C, indicating the feature role of W_2_C as an active component. This role was further validated by more negative onset potentials (<1.4 V versus SCE) for the electrochemical alkoxylation reaction on W_2_C/N_*x*_C anodes than that (>1.6 V versus SCE) of the bare carbon cloth electrode (Supplementary Fig. 23). However, the activation of adsorbed C–H bonds is enhanced by the electron-deficient surface of the W_2_C-0.07e^−^ model, as reflected by the more pronounced electron density difference (Hirshfeld charge) of the preadsorbed C–H bonds (Fig. 3c and Supplementary Fig. 24) and a much lower calculated adsorption energy for ethylbenzene (Fig. 3e). Such strong adsorption of ethylbenzene molecules over the electron-deficient W_2_C surface was then experimentally validated by the temperature-programmed desorption (TPD) analysis results (Fig. 3f), exhibiting gradually elevated adsorption capacities over those of more electron-deficient W_2_C/N_*x*_C samples with similar surface areas. It should be noted that the bare carbon support (NC sample in Fig. 3f) provides a low adsorption capacity, only 21% of the best-in-class W_2_C/N_3.0_C sample (Supplementary Fig. 25). More importantly, the electron deficiency-induced adsorption behavior of ethylbenzene on the final W_2_C/N_*x*_C-based anodes under a fixed bias in the electrochemical reactor was well expressed with the same trend in adsorption capacities (Fig. 3g) as that revealed by TPD results, making successive C–H dissociation process more favorable.

Indeed, the stronger interaction between preadsorbed ethylbenzene molecules and electron-deficient W_2_C significantly reduces the Gibbs free energy of each step of the whole alkoxylation reaction pathway (Fig. 3e). The dissociation of C–H bonds of ethylbenzene on the electron-deficient W_2_C catalyst (W_2_C–0.07e^−^ model) is the rate-limiting step with a free energy change of only 0.34 eV, and the subsequent coupling of *C_8_H_9_ and *CH_3_O (*C_8_H_9_ + *CH_3_O) and desorption of as-formed (1-methoxyethyl)benzene (*C_9_H_12_O) proceed automatically. With similar configurations, the last three steps for the catalytic conversion of preadsorbed ethylbenzene molecules on the pristine W_2_C catalyst (W_2_C model) are thermodynamically uphill with a larger free energy change of 0.4 eV for the (*C_8_H_9_ + *CH_3_O) step, again indicating the key role of electron density in facilitating the whole reaction and desorption processes on the W_2_C surface. This electron-deficiency-dependent promotion effect on the activity of W_2_C was then unambiguously confirmed by the gradually increased catalytic activities (Fig. 3h) and *F*_E_ values (Fig. 3i) for producing (1-methoxyethyl)benzene on more electron-deficient W_2_C/N_*x*_C-based anodes under fixed work potential.

### Stability test and substrate scope

**Fig. 4:**
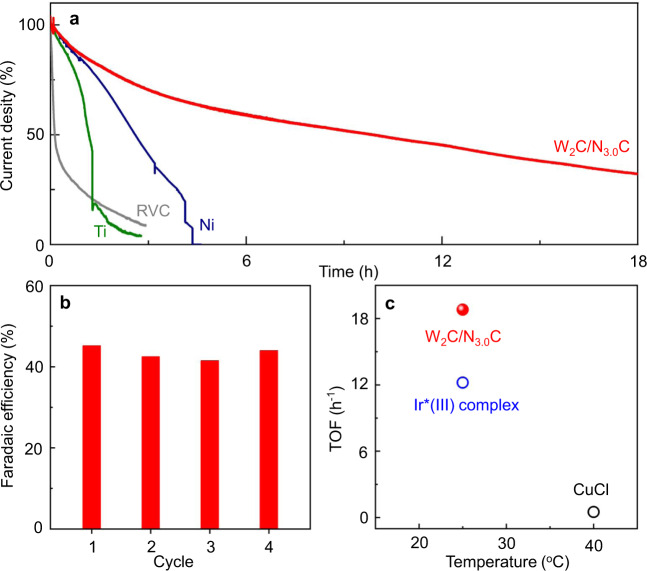
Stability and efficiency of C–H alkoxylation. **a** Current density change of W_2_C/N_3.0_C, Ti mesh, Ni plate, and RVC electrodes during the reaction. **b** Reusability of W_2_C/N_3.0_C under standard conditions within 3 h. **c** TOF values (for details, please see Supplementary Table 2) for methoxylation of benzylic C–H bonds on W_2_C/N_3.0_C-based heterogeneous system (solid sphere) and by state-of-the-art photocatalyst (Ir*(III) complex, blue circle) and homogeneous catalyst (CuCl, black circle).

The W_2_C/N_3.0_C anode also shows excellent electrochemical stability. The composition (Supplementary Fig. 26) and morphology (Supplementary Fig. 27) of the used W_2_C/N_3.0_C materials were maintained well. Most importantly, the W_2_C/N_3.0_C anode can be recycled at least four times without an obvious decrease in *F*_E_ (41−46%) (Fig. 4b and Supplementary Fig. 28). It should be noted that inert anodes for alkoxylation of ethylbenzene, including carbon electrode (exemplified by RVC), stable metals (exemplified by Ti mesh), and active metals (exemplified by Ni plate), decompose rapidly within 5 h (Fig. 4a and Supplementary Fig. 29), illustrating the key importance of the high activity of the W_2_C/N_3.0_C anode to keep itself from corroding. As the best-in-class anode in this work, the W_2_C/N_3.0_C electrode provides a high turnover frequency (TOF) value of 18.8 h^−1^, which is comparable to or even higher than the reported values, mostly of homogeneous catalysts, for similar alkoxylation reactions (Fig. 4c and Supplementary Tables 2–4)^43,44^.

**Fig. 5:**
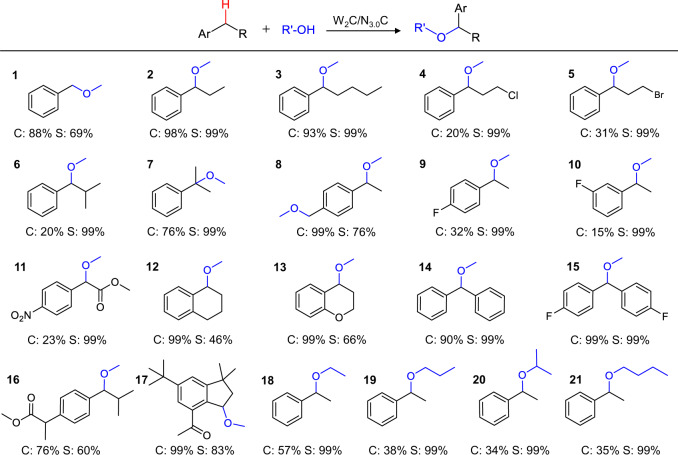
Scope of substrates catalyzed by W_2_C/N_3.0_C anode. Standard reaction conditions: **1**-**15**, substrate (0.5 mmol), lithium perchlorate (1 mmol), methanol (15 mL), W_2_C/N_3.0_C anode, and Ti mesh cathode with a controlled potential of 2.0 V versus SCE reference electrode for 18 h at room temperature. **16** and **17**, bioactive molecules (0.25 mmol) for 18 h and 4 h, respectively. **18**–**21**, ethylbenzene (0.5 mmol), alcohols (15 mL), W_2_C/N_3.0_C anode, and Ti mesh cathode with a controlled potential of 2.0 V versus SCE reference electrode for 18 h at room temperature.

As a durable anode, the electron-deficient W_2_C electrode exhibits satisfying activity for electrochemical alkoxylation of various aromatic C–H bonds using a series of aliphatic alcohols (Fig. 5) with good to high conversions and excellent selectivity. Toluene was methoxylated to (methoxymethyl)benzene with high conversion (**1**). Benzenes with alkyl chains, including propylbenzene, pentylbenzene, isobutylene, and even halide-substituted substrates (**2**–**6**), were successfully transformed into target products with good conversions (20–98%) and very high selectivity (>99%). Alkoxylation of tertiary C–H in cumene (**7**) could also proceed smoothly with the alkoxylation product as the sole product. Alkylbenzenes with electron-withdrawing groups (**8**–**11**) were also tolerated on the electron-deficient W_2_C-based anode for highly selective alkoxylation reactions even with high steric hindrance. Tetralin and chroman (**12** and **13**), as substructures present in numerous drugs, underwent effective methoxylation. It is also not surprising that benzhydryls (**14** and **15**) with activated C–H

bonds could be rapidly transformed into alkoxylation products under standard conditions with very high selectivity (>99%). Besides, electrochemical methoxylation could also apply for the late-stage modification of bioactive molecules. For example, celestolide (**16**) and ibuprofen methyl ester (**17**) were able to couple with methanol and shown excellent selectivity at benzylic position. Electron-deficient W_2_C-based anode was also powerful for the alkoxylation of ethylbenzene with different types of aliphatic alcohols (**18**-**21**). All these results again reveal the efficiency of the electron-deficient W_2_C catalyst for the benzylic C–H bonds alkoxylation with excellent chemical tolerance to various functional groups.

## Discussion

In this work, we have demonstrated the key role of electron-deficient W_2_C nanocrystals as electrode materials in boosting the activity and durability for electrochemical activation of C–H bonds via a heterogeneous pathway. We successfully tuned the electron density of W_2_C nanocrystals by constructing Schottky heterojunctions with nitrogen-doped carbons to achieve preferred adsorption of benzylic C–H bonds of ethylbenzene on the W_2_C surface and facilitate subsequent C–H activation, which is the rate-limiting step. Unlike conventional oxidative alkoxylation to generate water, the as-formed protons on the W_2_C anode could be simultaneously converted to hydrogen gas in our additive-free electrochemical reactor under mild conditions. This two-birds-with-one-stone strategy illustrates the significant potential of powerful designer electrode materials to substantially increase catalytic efficiency, atomic economy, and electric energy utilization for organic electrosynthesis and hydrogen energy production in one electrocatalytic system. In addition to the hydrogen evolution reaction, the reduction process might be compatible with other important reactions (e.g., carbon dioxide reduction reaction or N_2_/NO_x_ reduction reactions) to create more complex cascade reaction pathways for the production of high value-added compounds from abundant hydrocarbons and even waste gases. The electron deficiency shows great potential to enhance the intrinsic activity of catalysts in various catalytic systems, and may also boost the development of zero-additive and zero-emission electrosynthesis systems. We envision that the practical application of the heterojunction-based electrode materials for organic synthesis, commercialized nanocarbons (e.g., nitrogen-doped carbon spheres, carbon nanotube, and even graphenes) could be merged together with functional metal nanoparticles on the surface of various carbon cloth and metal meshes in flow cells for continuous production.

## Methods

### Synthesis of W_2_C/N_*x*_C

All chemicals were analytical grade and used as received without further purification. A homogenous mixed solution including ammonium tungstate ((NH_4_)_6_H_2_W_12_O_40_·nH_2_O, 0.75 g), dicyandiamide (DCDA, 15 g), and deionized water (150 mL) was heating at 80 °C, and the mixed solution was evaporated at a constant temperature with stirring. The obtained mixture after evaporating water was transferred into a cylindrical crucible with a lid and heated at 1000, 1100, and 1200 °C for 2 h with a heat rate of 2.5 °C min^−1^ under a high purity nitrogen atmosphere. The final products after cooling down to room temperature were named W_2_C/N_*x*_C (*x* represents the nitrogen contents) and used for further experiments and characterizations.

The control group of nitrogen-doped carbon (NC) was prepared with the same procedure as W_2_C/N_*x*_C without metal precursors. And W_2_C and NC mixture was obtained by mixing W_2_C powder and NC mechanically.

### Materials characterization

Powder X-ray diffraction patterns (XRD) was performed using a Bruker D8 Advance X-ray diffractometer equipped with a Cu Kα radiation source (λ = 1.5418 Å) and operated at a scan rate of 6° min^−1^. Scanning electron microscopy (SEM) was operated on FEI Nova NanoSEM 450 field emission scanning electron microscope. Transmission electron microscopy (TEM), high-resolution transmission electron microscopy (HRTEM), and energy-dispersive X-ray (EDX) analysis were measured on a JEM-2100F microscope with an acceleration voltage of 200 kV. Temperature programmed desorption (TPD) was carried out on a Micromeritics Autochem II chemisorption analyzer with ethylbenzene probe molecules at 110 °C. Nitrogen adsorption-desorption isotherms were acquired on Quantachrome NOVA-2200e at 77 K. Prior to the measurement, the samples were degassed at 200 °C for 12 h with a gas flow of nitrogen. X-ray photoelectron spectroscopy (XPS) and ultraviolet photoelectron spectroscopy (UPS) experiments were recorded at a Kratos Axis Ultra DLD spectrometer and ESCALAB 250 photoelectron spectrometer (Thermo Fisher Scientific), respectively. The inductively coupled plasma atomic emission spectroscopy (ICP-AES) measurement was conducted on an iCAP6300 spectrometer for tungsten element analysis.

### Electrochemical measurements

All of the electrochemical experiments were performed in a standard three-electrode system on an electrochemical station (CHI 660E, Shanghai CH Instruments Company). Working electrodes were composed of catalysts supported by carbon cloth. The catalyst ink was prepared by sonicating and dispersing 5 mg of catalyst into a solution containing 700 μL of ethanol, 350 μL of deionized water, and 160 μL of 5% Nafion solution. The working electrodes were prepared by evenly dipping 150 μL of ink onto carbon cloth (1 × 1 cm) and dried at 120 °C for 1 h in the oven. Titanium (Ti) mesh with a size of 1 × 1 cm and saturated calomel electrode (SCE) were employed as counter and reference electrodes, respectively. The control electrodes with different sample loading were prepared by dipping 50, 100, and 200 μL of ink on carbon cloth, respectively. The electrocatalytic reactions were conducted in 15 mL of methanol with 61 μL of ethylbenzene (0.5 mmol) and 0.106 g of lithium perchlorate (1 mmol) at room temperature in a home-made electrolyzer in which methanol was not only used as a solvent but also simultaneously used as a reactant. The electrocatalytic stability tests of catalyst for the reaction between ethylbenzene and methanol were evaluated using the same reaction potential for four consecutive cycles, and electrodes would have to dry at 120 °C before the next reaction.

Electrode adsorption experiments were conducted in an electrolyzer containing 1.5 mmol of ethylbenzene, 15 mL of methanol, and 1 mmol of lithium perchlorate with W_2_C/N_x_C anodes, Ti mesh cathode, and saturated calomel electrode. To accurately measure the adsorption volume of ethylbenzene before transforming (1-methoxyethyl)benzene, we performed at 2 V versus SCE for 10 min. After the completion of adsorption, the residual volume of ethylbenzene in solution was obtained by extraction and analyzed on gas chromatography-mass spectrometry (GC-MS, Shimadzu QP2010SE) with dodecane as internal standard. And the adsorption volume for ethylbenzene on W_2_C/N_*x*_C (C_ads_) was calculated using the equation of *n*_ads_ = *n*_ini _− n_res_ (*n*_ini_ and *n*_res_ represent the initial and residual contents in solution, respectively)^45^.

### Products analysis

After the reaction, 500 μL of the solution was firstly taken from electrolyzer and transferred into an extracted solution containing 500 μL of dichloromethane, 500 μL of deionized water and 0.2 μL of dodecane, and then dried by magnesium sulfate anhydrous. Finally, the extracted solution was analyzed in GC-MS to determine the components of products and calculate the conversion and selectivity.

The Faradaic efficiency (*F*_E_) was calculated as follow:

1 $${{{F}}}_{{\rm{E}}}=\frac{{{{N}}}_{i}\times {{n}}\times {{F}}}{{{Q}}}$$

where *N*_*i*_ is the number of moles for the specific product (mole); *n* is the number of electrons exchanged for product formation, which is 2 e in this reaction; *F* is the Faradaic constant of 96487 C mol^−1^; *Q* is the passed charge.

Turnover frequency (TOF) was defined by the following equation:

2 $${\rm{TOF}}=\frac{{{n}}}{{{N}}\times {{h}}}$$

where *n* is the number of moles for the product; *N* is the number of moles of active metal sites determined from ICP-AES.

### Theoretical calculation

The spin polarization density functional theory (DFT) calculations were performed by the DMol3 program on Materials Studio. The generalized gradient approximation method with Perdew-Burke-Ernzerhof functional (GGA-PBE) was used for describing the exchange-correlation interaction among electrons^46^. The double numerical plus polarization (DNP) basis set was employed, while an accurate DFT semi-core pseudopots (DSPP) was adapted to describe the metal atoms^47,48^. The 6 × 6 × 1 k-points were used for sampling the Brillouin zone. Hexagonal W_2_C (002) facets were modeled in terms of the slabs of 5 × 5 supercells with 14.76 × 14.76 Å and 120^o^ and W_2_C cluster model was placed above a 6 × 6 supercell of graphene lattice^49^. The vacuum slab was set as 20 Å to calculated all periodical models. The structure of the carbon support models was constructed on the basis of concentrations of pyridinic N and graphitic N dopants in the carbon lattice from the XPS analysis results. It should be noted that the bond lengths of small cluster models were fixed during the calculations. The charge exchange was calculated based on a small W_4_C_2_ cluster and NC support. The mean number of 0.07 electrons per atom (Supplementary Fig. 12) according to the W_4_C_2_ cluster/N_3.0_C model was used for following the construction of charged W_2_C slab model to study the interaction between reactant molecule and catalyst surface^50^. It should be noted that the small cluster models are not used for following the simulation of the catalytic process. For the W_2_C slab models, the crystal unit model with the selected (002) facet was used according to HRTEM (Fig. 1b) and XRD (Supplementary Fig. 10) analysis.

The Gibbs free energy change (Δ*G*) for each step of ethylbenzene activation was calculated as follows:

3 $$\Delta G=\Delta E+\Delta {{\mathrm{ZPE}}}-T\Delta S(T=298.15\;K)$$

where Δ*E*, ΔZPE, and Δ*S* are the changes in the reaction energy, zero-point energy, and entropy, respectively.

## Supplementary information

Supplementary Information

Movie

Description of additional supplementary files

## Data Availability

The data that support the findings of this study are available from the corresponding author upon reasonable request. Source data are provided with this paper.

## Source data

Source Data
