# Supplementary material for: Electrochemical activation of C–H by electron-deficient W2C nanocrystals for simultaneous alkoxylation and hydrogen evolution
Source: Nat Commun. 2021 Jun 23;12:3882. doi: 10.1038/s41467-021-24203-8 (PMC8222219; doi:10.1038/s41467-021-24203-8)
Supplement: Supplementary file 1 — Supplementary Information [file 41467_2021_24203_MOESM1_ESM.pdf]

Supplementary Information

Electrochemical activation of C–H by electron-deficient W_2C nanocrystals for simultaneous alkoxylation and hydrogen evolution

Lin et al.

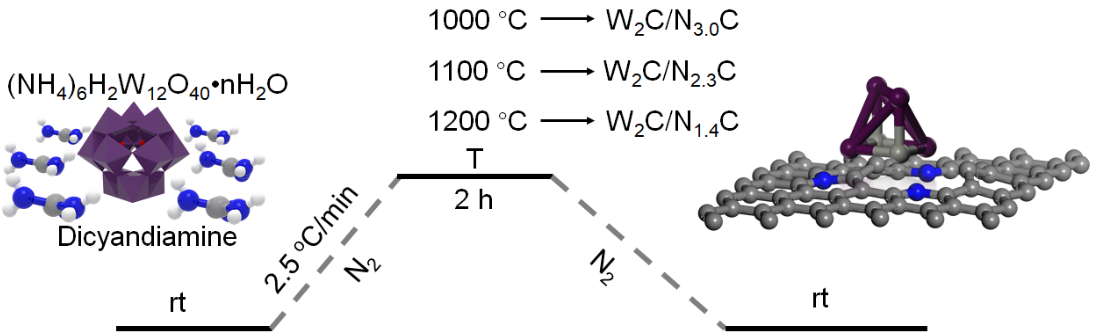

Supplementary Figure 1. Detailed synthetic route for the preparation of W_2C/N_xC samples, where x represents the nitrogen contents (at.%). Temperatures (T): 1000, 1100 and 1200 °C for $W_2C/N_{3.0}C$, $W_2C/N_{2.3}C$ and $W_2C/N_{1.4}C$, respectively; Colour codes: C, grey; N, blue; H, white; W, purple.

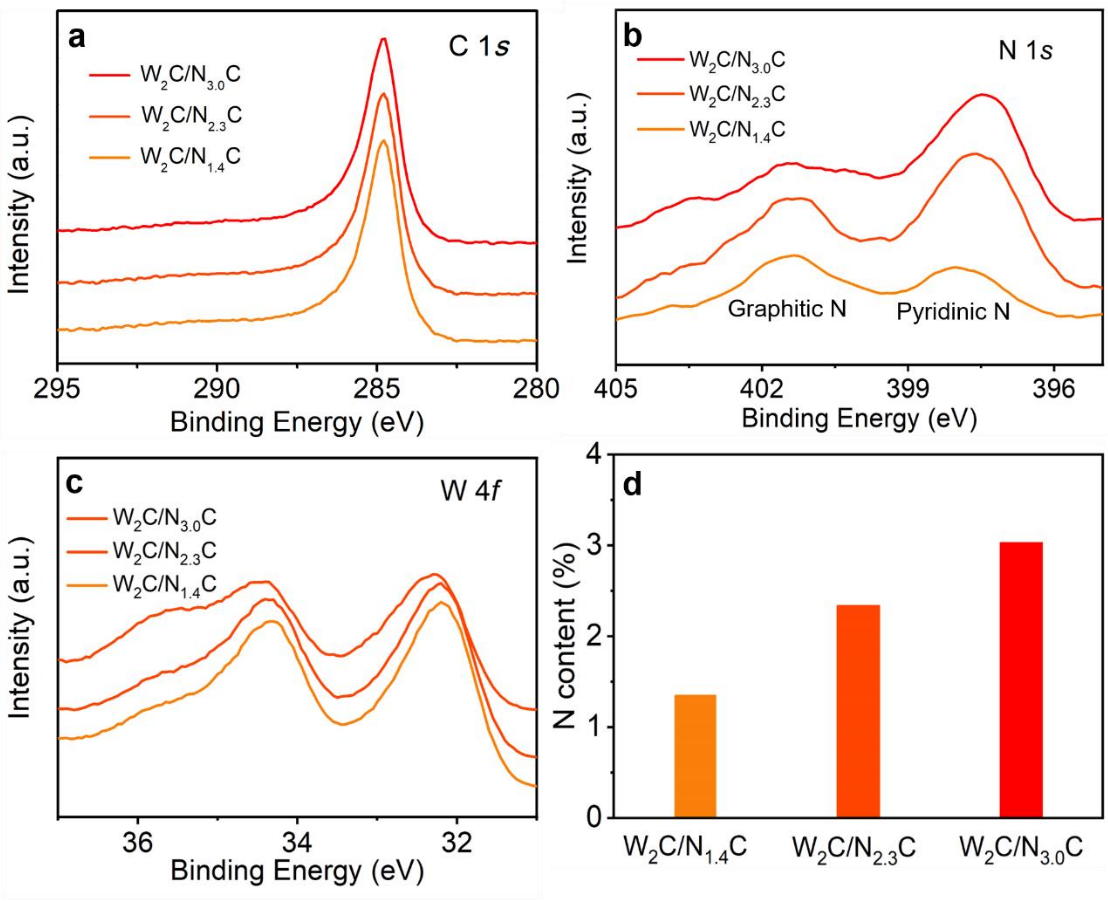

Supplementary Figure 2. High-resolution C 1*s* (**a**), N 1*s* (**b**) and W 4*f* (**c**) spectra of W_2C/N_xC samples. (**d**) Nitrogen contents estimated from the XPS analysis results. The N contents (at.%) are 3.0%, 2.3% and 1.4% for $W_2C/N_{3.0}C$, $W_2C/N_{2.3}C$ and $W_2C/N_{1.4}C$, respectively.

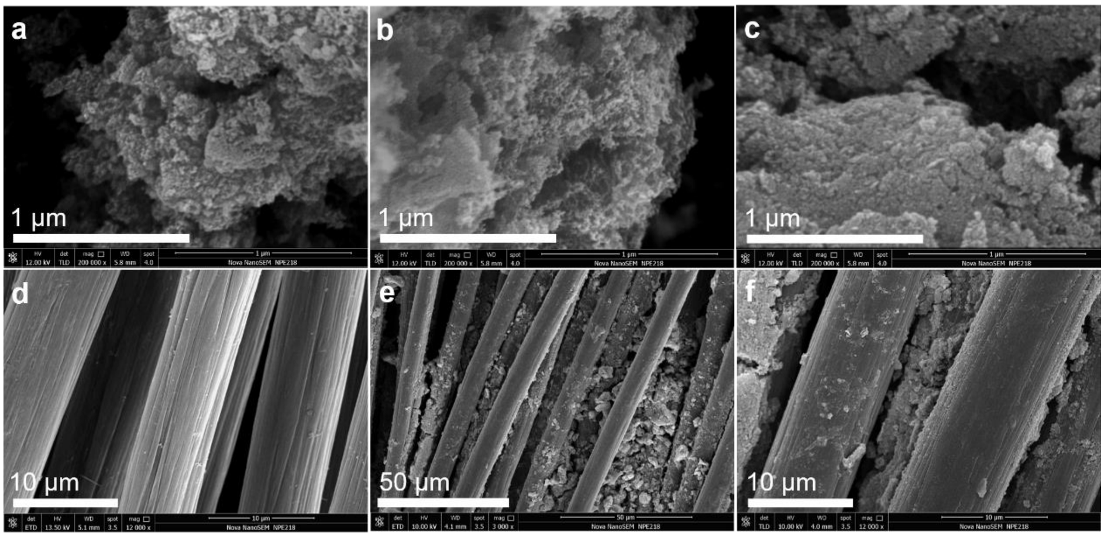

Supplementary Figure 3. (**a**-**c**) SEM images of $W_2C/N_{3.0}C$, $W_2C/N_{2.3}C$ and $W_2C/N_{1.4}C$ samples, respectively. All samples exhibit similar morphology with rough surfaces. SEM images of bare carbon cloth (**d**) and $W_2C/N_{3.0}C$ supported on bare carbon cloth (**e**,**f**).

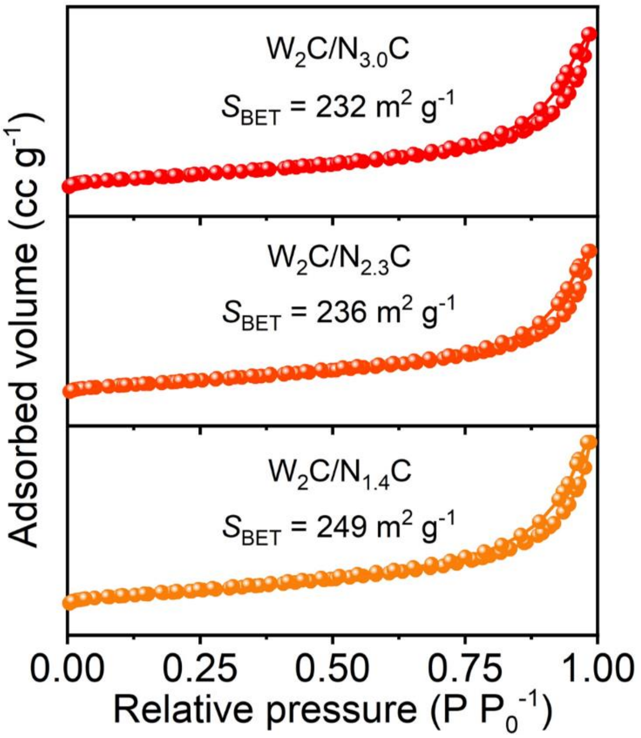

Supplementary Figure 4. N_2 adsorption-desorption isotherms and specific surface areas (S_{BET}) of W_2C/N_xC samples.

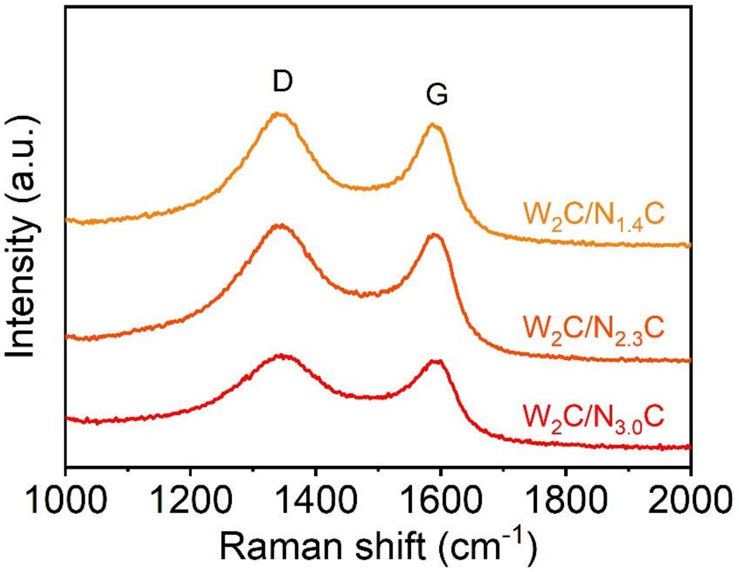

Supplementary Figure 5. Structural information of W_2C/N_xC was obtained from Raman spectra. The I_D/I_G values were determined to be 1.07, 1.04 and 1.03 of $W_2C/N_{3.0}C$, $W_2C/N_{2.3}C$ and $W_2C/N_{1.4}C$, respectively.

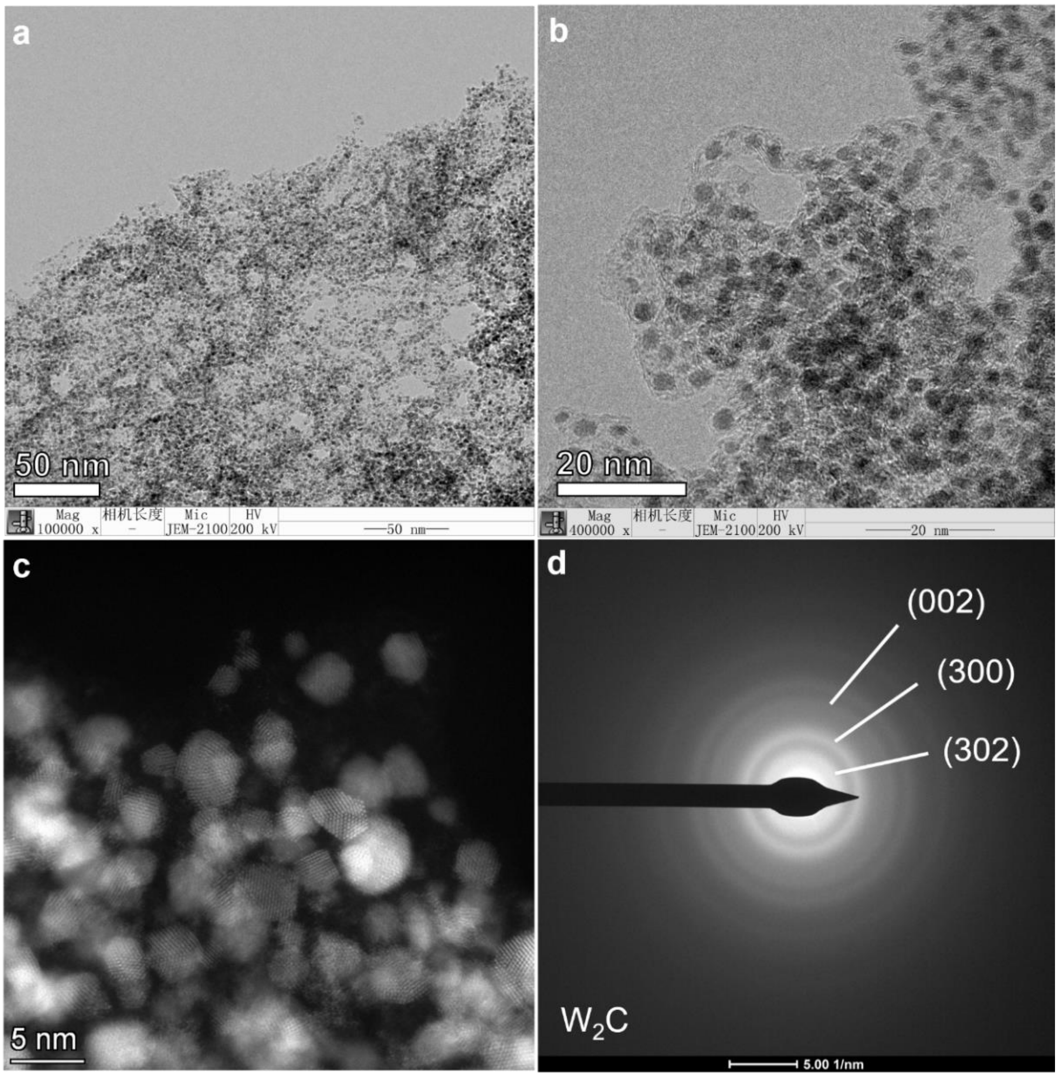

Supplementary Figure 6. (**a** and **b**) TEM images of $W_2C/N_{3.0}C$. W_2C particles disperse uniformly on few-layer carbon supports without obvious aggregation. (**c**) HAADF-STEM image of $W_2C/N_{3.0}C$. (**d**) Selected-area electron diffraction pattern illustrates the formation of W_2C nanocrystals.

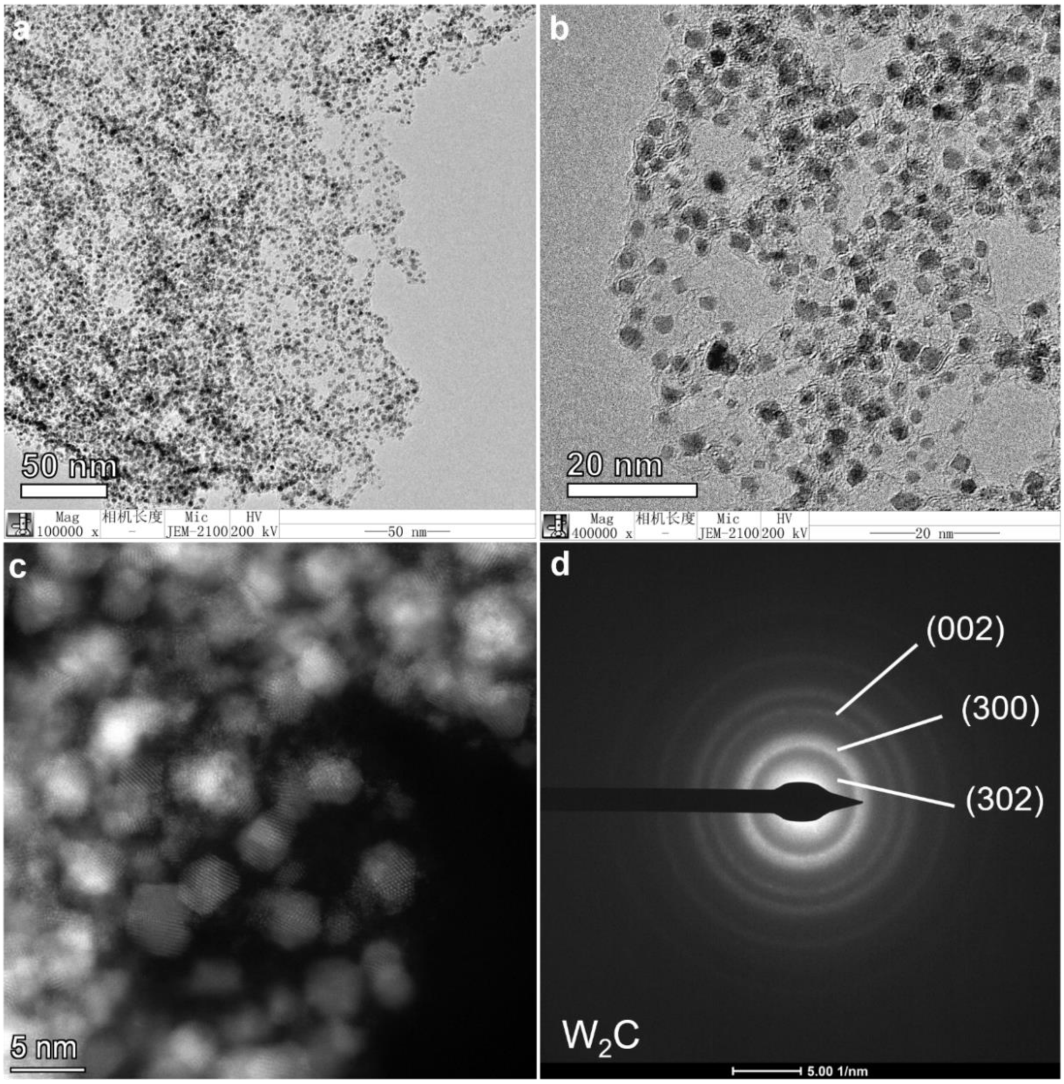

Supplementary Figure 7. (**a** and **b**) TEM images of $W_2C/N_{2.3}C$. (**c**) HAADF-STEM image of $W_2C/N_{2.3}C$. (**d**) Selected-area electron diffraction pattern illustrates the formation of W_2C nanocrystals.

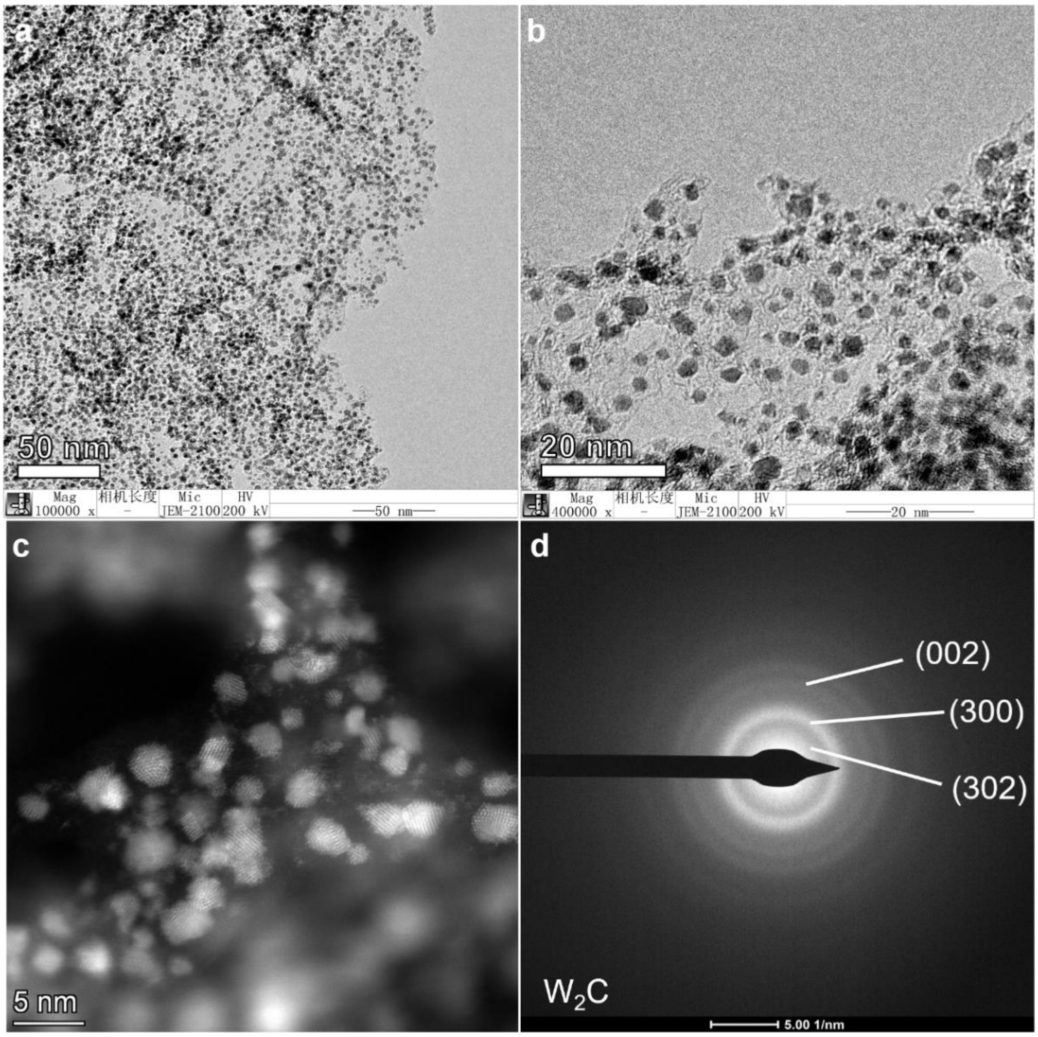

Supplementary Figure 8. (**a** and **b**) TEM images of $W_2C/N_{1.4}C$. (**c**) HAADF-STEM image of $W_2C/N_{1.4}C$. (**d**) Selected-area electron diffraction pattern illustrates the formation of W_2C nanocrystals.

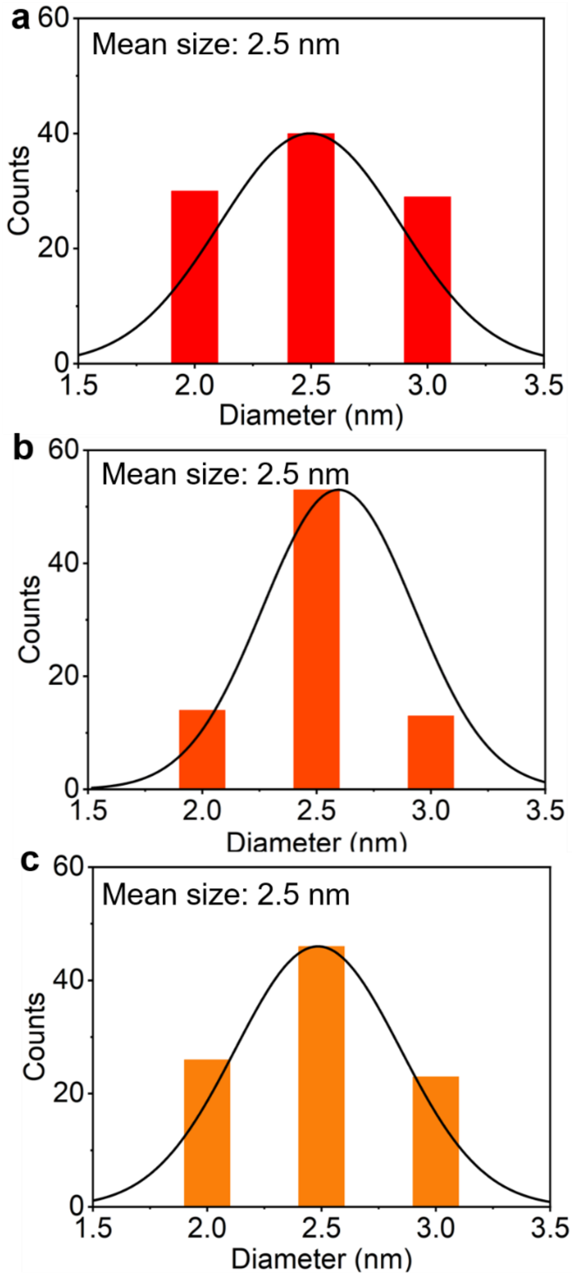

Supplementary Figure 9. The size distribution of $W_2C/N_{3.0}C$ (**a**), $W_2C/N_{2.3}C$ (**b**) and $W_2C/N_{1.4}C$ (**c**). The gradually elevated synthetic temperatures did not change the size distribution of these samples.

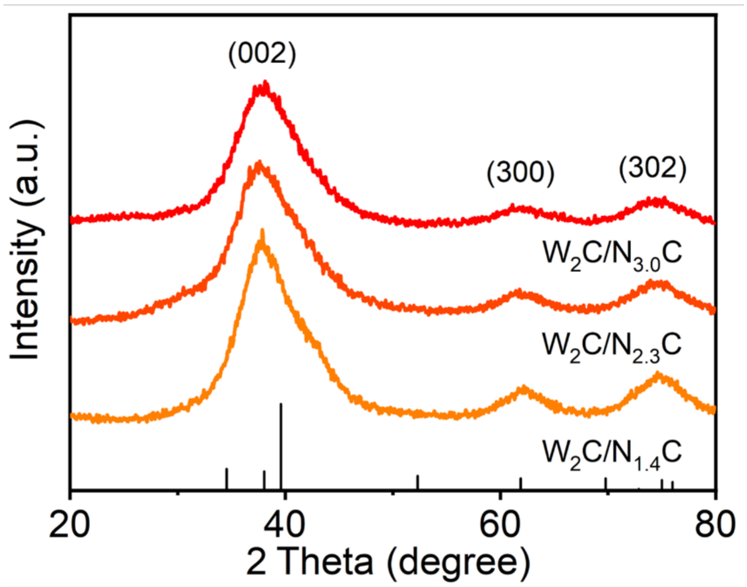

Supplementary Figure 10. XRD patterns of W_2C/N_xC samples. The XRD peaks of W_2C/N_xC samples could be attributed to the characteristic(002), (300) and (302) facets of α-W_2C (black bars, JCPDS no. 35-776).

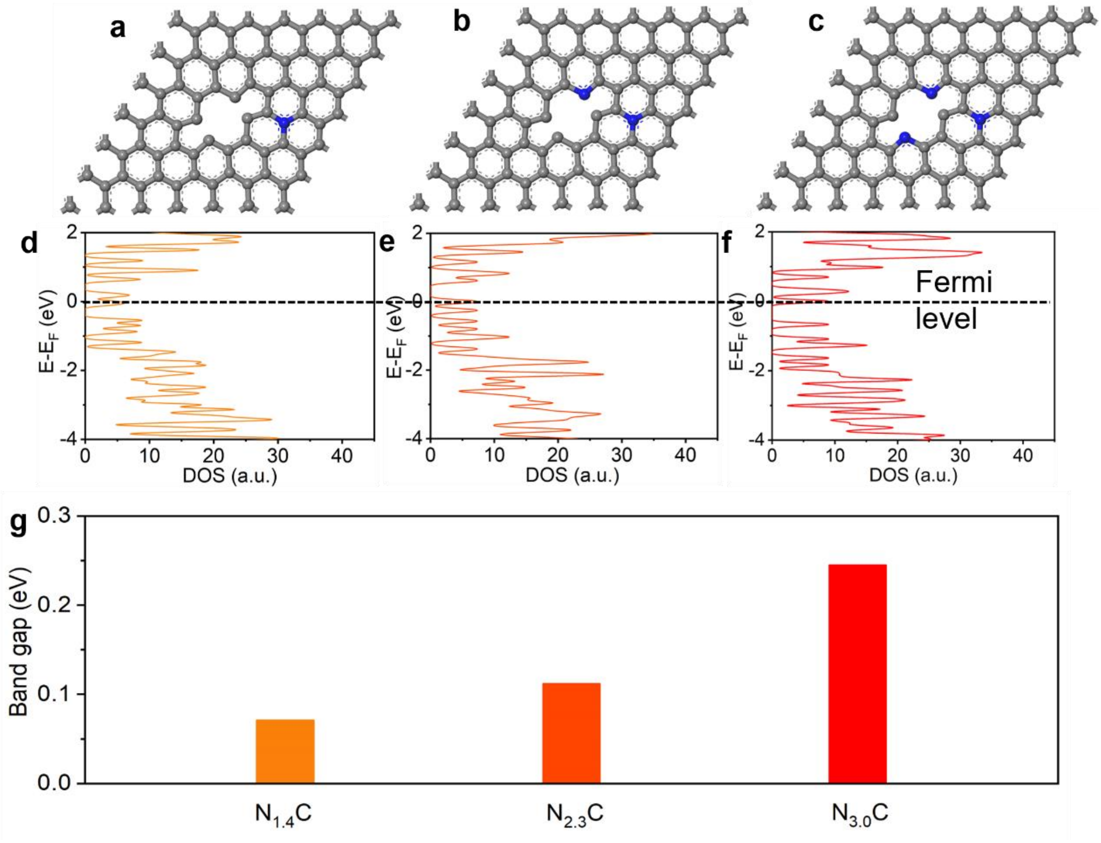

Supplementary Figure 11. Computation models and density of states of $N_{1.4}C$ (**a** and **d**), $N_{2.3}C$ (**b** and **e**) and $N_{3.0}C$ (**c** and **f**). Colour code: C, grey; N, blue. The structure of the carbon support models were constructed on the basis of concentrations of pyridinic N and graphitic N dopants in the carbon lattice from the XPS analysis results (Supplementary Fig. 2). (**g**) The band gaps of $N_{1.4}C$, $N_{2.3}C$ and $N_{3.0}C$ were 0.071, 0.112 and 0.245 eV, respectively. Doping more nitrogen atoms into the carbon support opened the band gap.

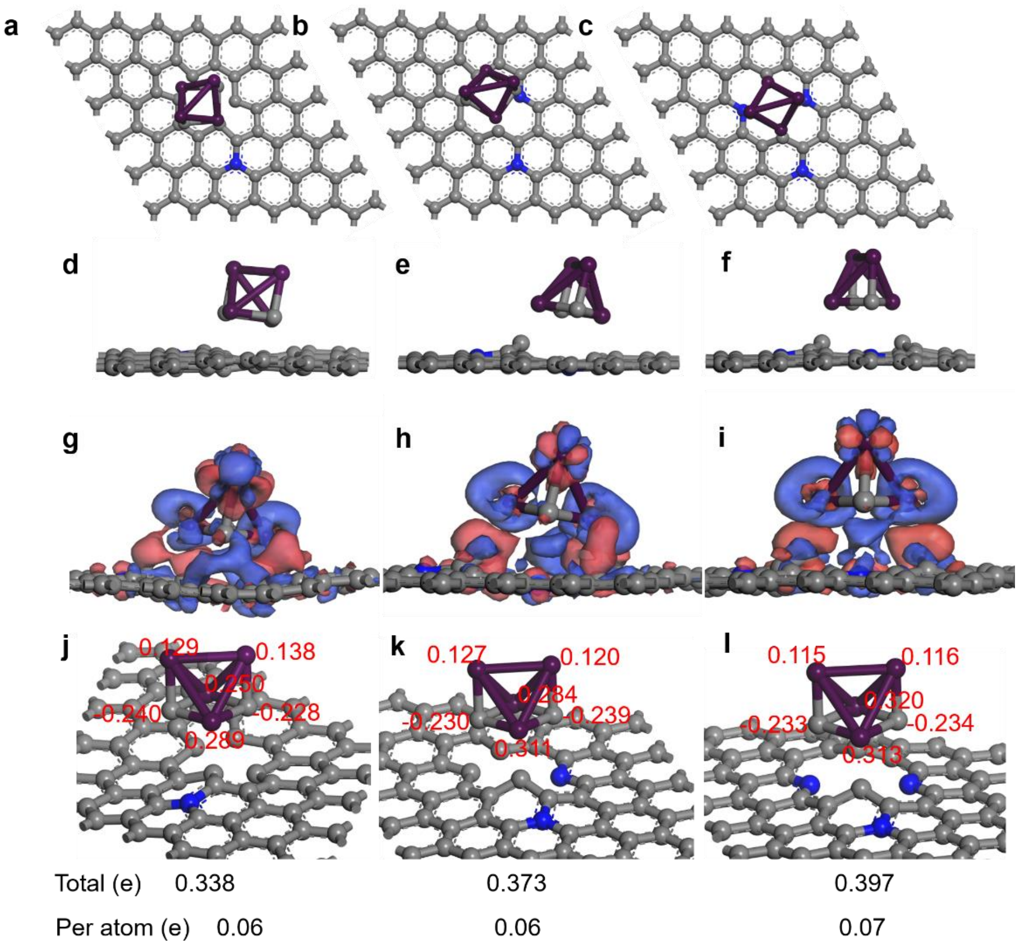

Supplementary Figure 12. The calculation models and electron density distribution of small cluser models. The top view and side view of $W_4C_2/N_{1.4}C$ (**a** and **d**), $W_4C_2/N_{2.3}C$ (**b** and **e**) and $W_4C_2/N_{3.0}C$ (**c** and **f**). Electron density difference stereograms of $W_4C_2/N_{1.4}C$ (**g**), $W_4C_2/N_{2.3}C$ (**h**) and $W_4C_2/N_{3.0}C$ (**i**) models (electron-deficient area, blue; electron-rich area, red); Colour code: C, grey; N, blue; W, purple. Quantify of charge exchange from W_4C_2 cluster to various nitrogen-doped carbon support for $W_4C_2/N_{1.4}C$ (**j**), $W_4C_2/N_{2.3}C$ (**k**) and $W_4C_2/N_{3.0}C$ (**l**) models. The methods to simulate the reaction on nanocatalysts are mainly based on the W_4C_2 cluster model with the same crystalline parameters[1] due to the same morphology of W_2C nanoparticles in our W_2C/N_xC samples (Supplementary Fig. 6-8). It should be noted that the bond lengths of small W_4C_2 cluster model was fixed during the calculations. From the aspect of metal-semiconductor heterojunction, the relaxed model does not maintain the metallic properties of W_2C crystal for simulating the essential metal-support effect (Mott-Schottky effect) due to structure distortion.

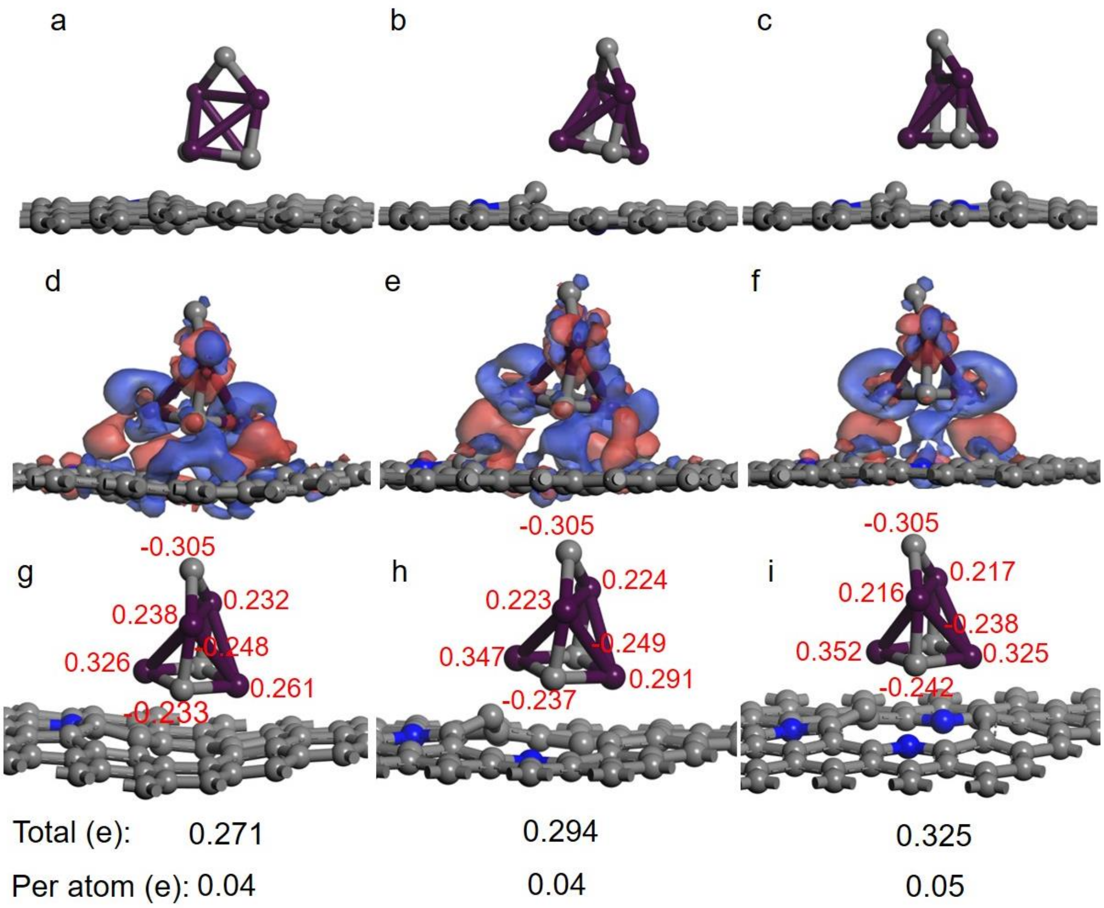

Supplementary Figure 13. The effect of cluster stoichiometry. The calculation models, electron density distribution and electron transfer numbers of $W_4C_3/N_{1.4}C$ (a,d,g), $W_4C_3/N_{2.3}C$ (b,e,h), and $W_4C_3/N_{3.0}C$ (e,f,i). We found the same trend of electron donation from the W_4C_3 cluster (0.04-0.05 electrons per atom) to the NC support, comparable to that of W_4C_2 cluster (0.06-0.07 electrons per atom) (Supplementary Fig. 12).

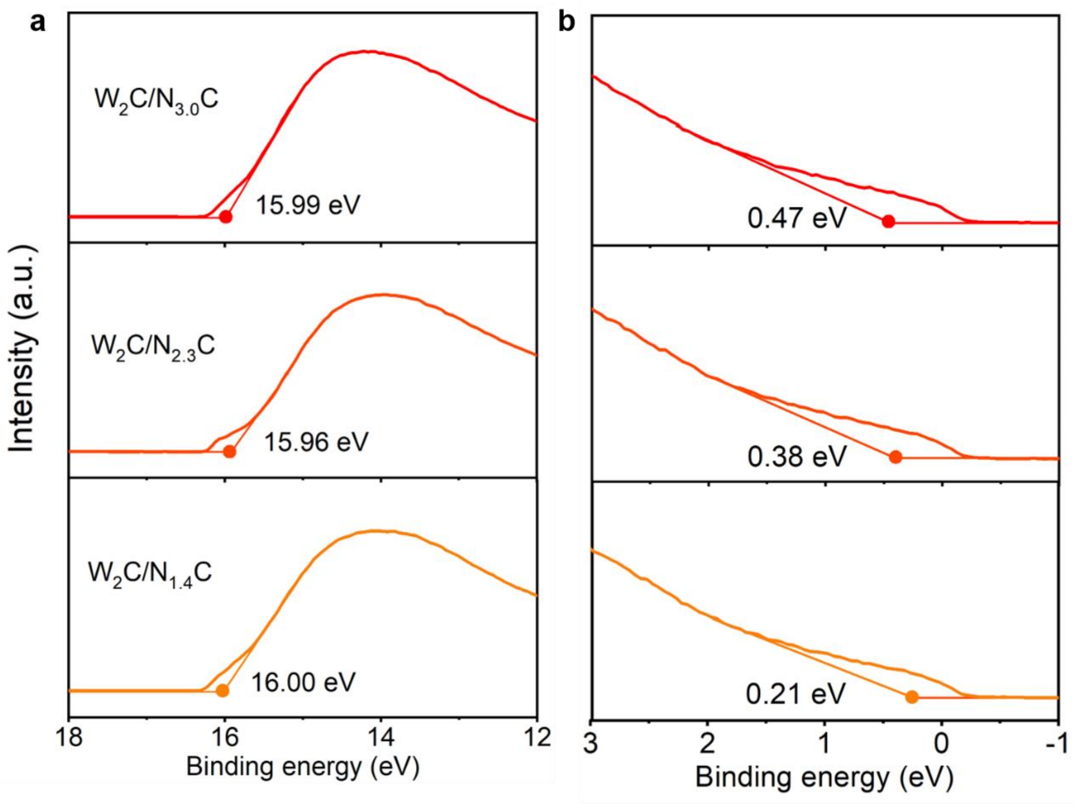

Supplementary Figure 14. UPS patterns of typical W_2C/N_xC samples. Secondary electron cut-off ($E_{cut\text{-}off}$) (**a**) and onset (E_F) energy (**b**). The work function (Φ), as calculated by the equation of $\Phi = 21.21$ eV - $(E_{cutoff} - E_F)^2$, are 5.69, 5.63 and 5.42 eV for $W_2C/N_{3.0}C$, $W_2C/N_{2.3}C$ and $W_2C/N_{1.4}C$ samples, respectively.

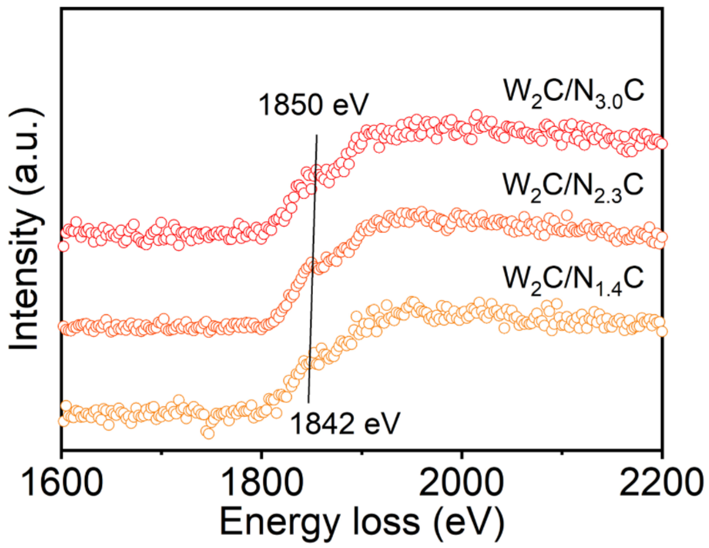

Supplementary Figure 15. EELS spectra of W_2C/N_xC samples. The W M4,5 peaks shift positively with the increase of nitrogen contents, which indicates that more electrons flowing from W_2C nanocrystals to nitrogen-doped carbons.

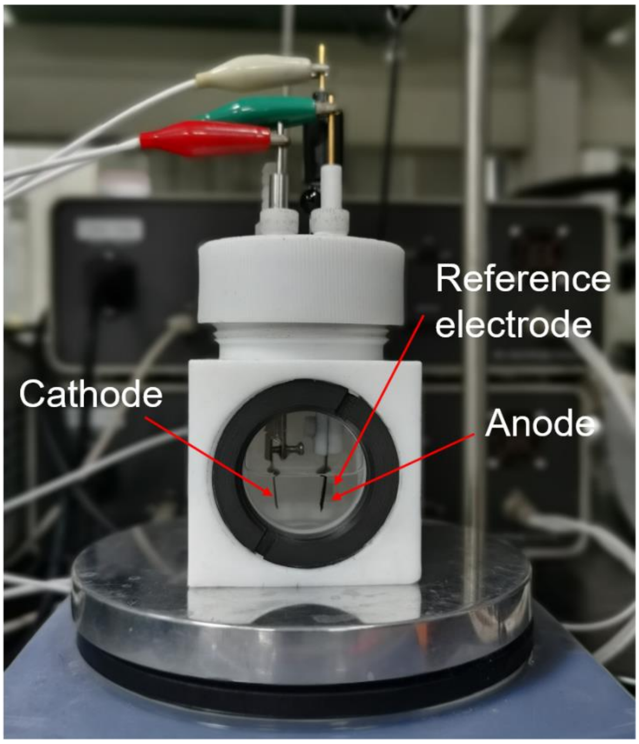

Supplementary Figure 16. Photograph of the reactor for electrochemical alkoxylation. A home-made reactor, composed of W_2C/N_xC anodes, Ti cathode and saturated calomel electrode (SCE) reference electrode, was used in this reaction system at room temperature. The electrolyte consisted of methanol (15 mL), ethylbenzene (0.5 mmol) and lithium perchlorate (1 mmol).

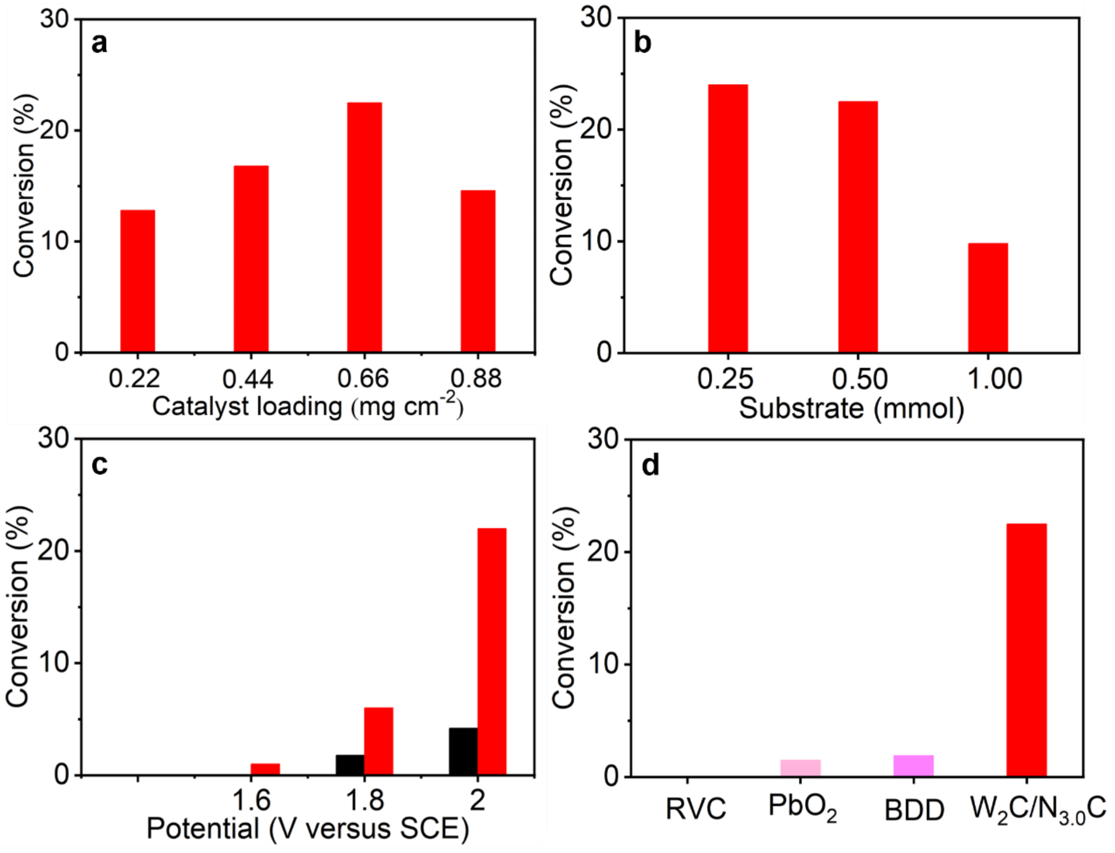

Supplementary Figure 17. Optimation of reaction conditions of $W_2C/N_{3.0}C$ catalyst. Conversions of (1-methoxyethyl)benzene with different catalyst loadings (**a**), substrate contents (**b**) and potentials (**c**) for the alkoxylation of ethylbenzene. According to the results, 0.66 mg/cm^2 of catalysts, and 0.5 mmol of ethylbenzene were used as the optimal parameters for all subsequent reactions at 2.0 V within 3 h. (**d**) The conversion of $W_2C/N_{3.0}C$ and common commercial electrodes. The outstanding activity of $W_2C/N_{3.0}C$ compared to the common commercial electrodes further demonstrates the significance of electron-deficient W_2C nanocrystals in boosting alkoxylation of C–H bonds.

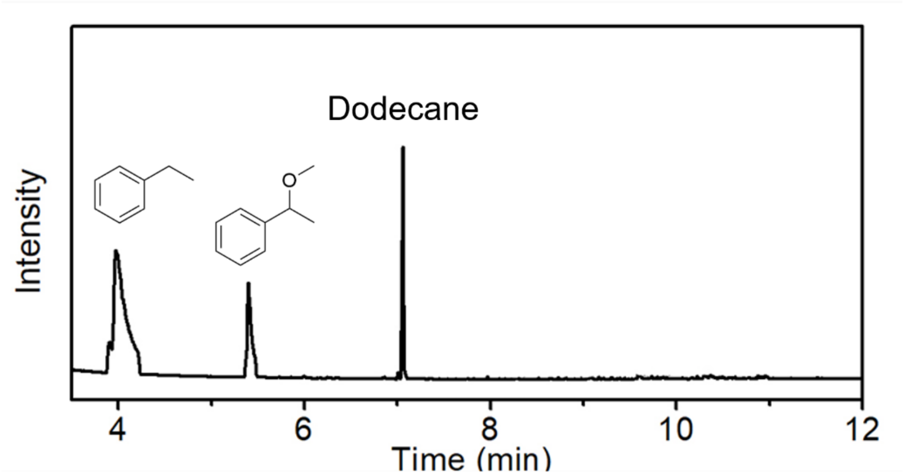

Supplementary Figure 18. Gas chromatography-mass spectrometry (GC-MS) spectrum of the electrolyte after electrochemical alkoxylation over the $W_2C/N_{3.0}C$ for 3 h. (1-methoxyethyl)benzene as the only product demonstrates the excellent selectivity. Dodecane was used as the internal standard for quantification.

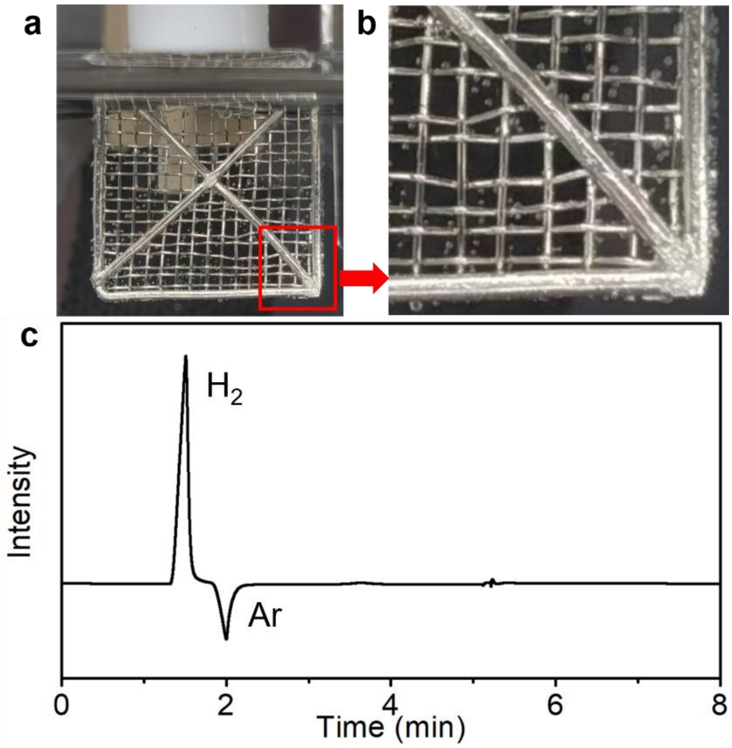

Supplementary Figure 19. Qualitative analysis of hydrogen. (**a** and **b**) The formation of hydrogen gas bubbles on the arbitrary cathode (exemplified with Pt mesh, please see Supplementary Video 1). (**c**) Gas chromatography (GC) spectrum of the collected gas during the alkoxylation reaction. Only hydrogen signal was detected in Ar carrier gas. GC analysis results are also used for following quantification of H_2 production.

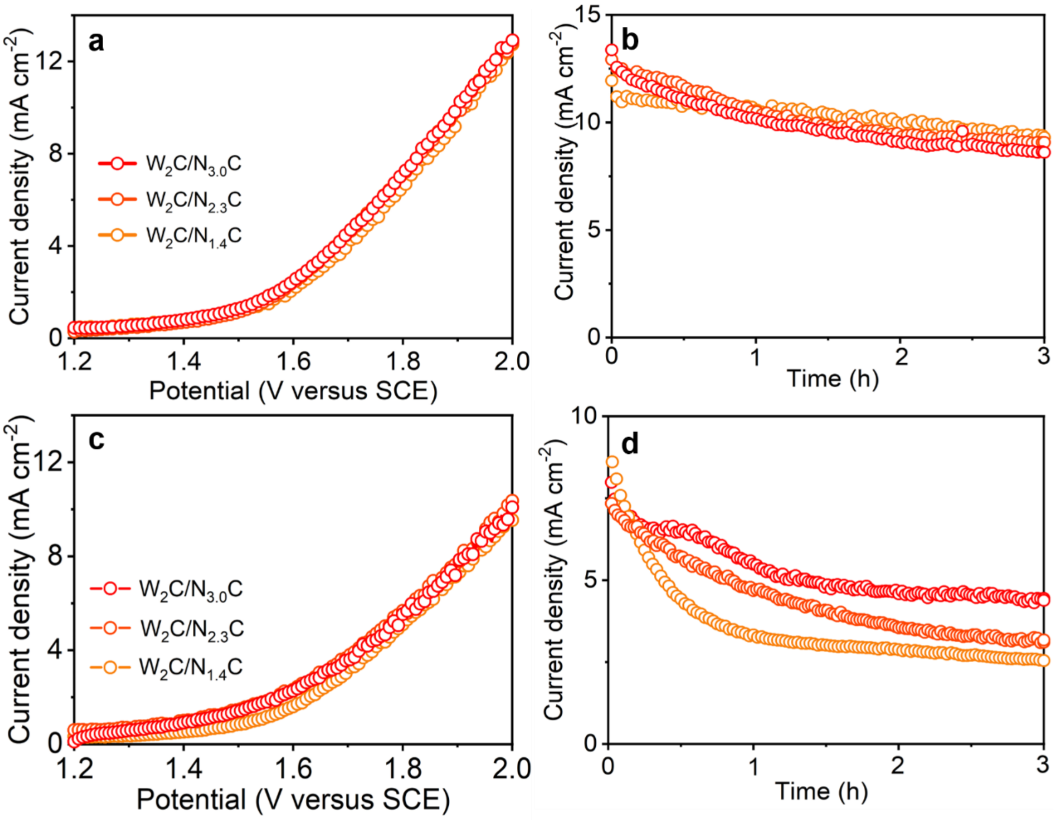

Supplementary Figure 20. Linear sweep voltammetry and chronoamperometric curves of W_2C/N_xC samples in the presence of ethylbenzene (**a** and **b**) and the absence of ethylbenzene (**c** and **d**) in the electrolyte.

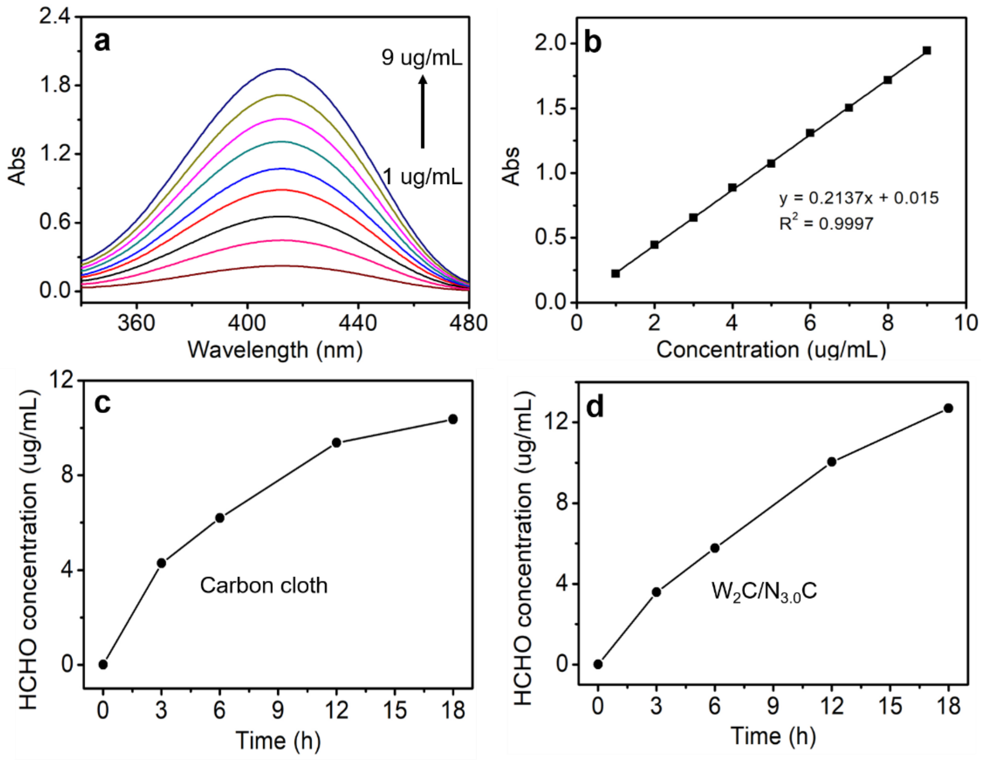

Supplementary Figure 21. Quantitative analysis of formaldehyde. (**a**) UV-vis absorption curves of standard solutions with known formaldehyde concentrations. The standard solutions consisted of different concentrations of formaldehyde and fixed amounts of acetylacetone solutions containing ammonium acetate (0.25 g), acetic acid (0.03 mL), acetylacetone (0.004 mL) and deionized water (2 mL). And then the pH of the solution was tuned to 6 by sulphuric acid (0.5 M). (**b**) Calibration curve used for the determination of formaldehyde at 413 nm. Time course of formaldehyde concentration on bare carbon cloth (**c**) and $W_2C/N_{3.0}C$ (**d**) anodes, respectively, generating a trace amount of formaldehyde (5 and 6 μmol, respectively) within 18 h, whilst 0.5 mmol of ethylbenzene was dehydrogenated and then transformed into (1-methoxyethyl)benzene. This observation excludes the obvious contribution of direct dehydrogenation reaction of methanol to formaldehyde to the total F_E for hydrogen evolution reactions.

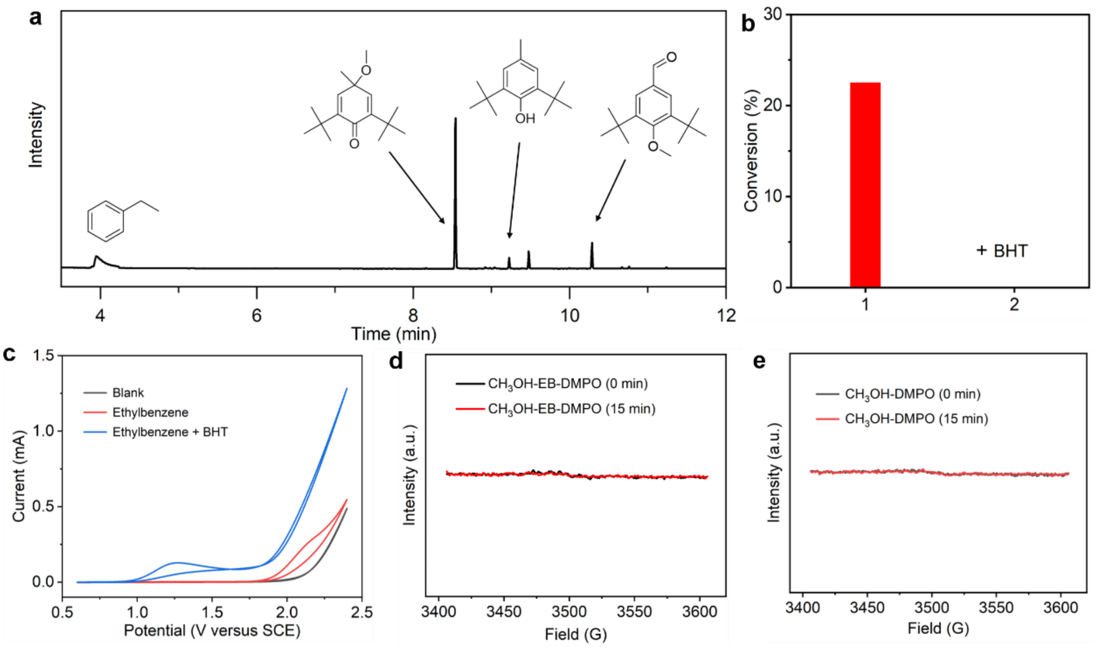

Supplementary Figure 22. (**a**) GC-MS spectrum of reaction solution with butylated hydroxytoluene (BHT). (**b**) Conversions of ethylbenzene over $W_2C/N_{3.0}C$ anode under standard reaction conditions (**1**) and in the control reaction with the addition of BHT (0.1 mmol) (**2**). After the addition of BHT, 2,6-ditert-butyl-4-methoxy-4-methyl-2,5-cyclohexadien-1-one and 3,5-di-tert-butyl-4-methoxybenzaldehyde with retention time of 8.6 min and 10.3 min were observed. Standard reaction conditions: ethylbenzene (0.5 mmol), lithium perchlorate (1 mmol), methanol (15 mL), $W_2C/N_{3.0}C$ anode and Ti mesh cathode with a controlled potential of 2.0 V versus SCE for 3 h at room temperature. (c) Cyclic voltammetry curves at 50 mV/s in a MeOH with $LiClO_4$ using a glassy carbon working electrode (5 mm^2), Ti mesh counter electrode, and SCE reference electrode. The EPR spectra of electrolytes including (d) methanol and (e) methanol and ethylbenzene in the same conditions. 0 and 15 min represent the addition times of DMPO (100 mM) after the reaction. A low potential oxidation peak was observed and no observable signals of any possible radicals were detected, indicating that BHT was possibly first oxidized and followed by addition with methanol.

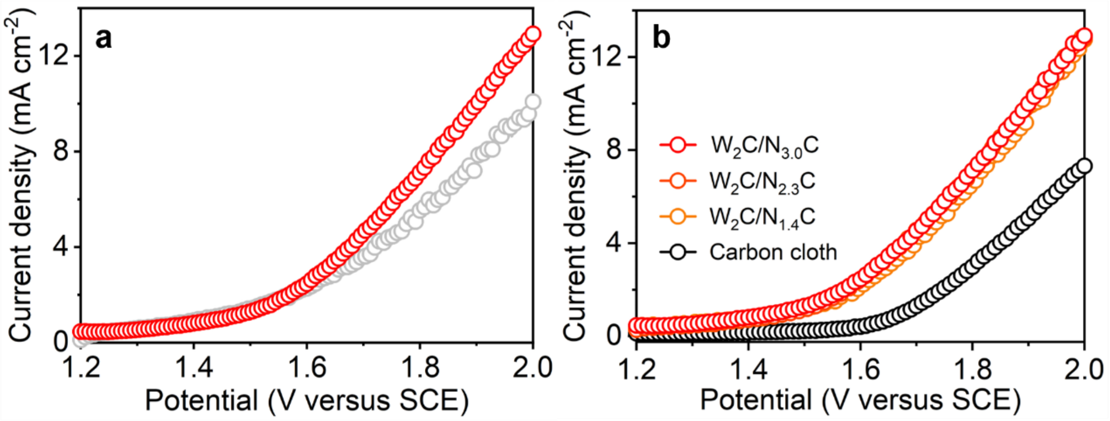

Supplementary Figure 23. (**a**) Linear sweep voltammetry curves of $W_2C/N_{3.0}C$ anode before adding ethylbenzene (grey) and after adding ethylbenzene (red) under standard reaction conditions with a scanning rate of 10 mV/s. The enhanced current density after the addition of ethylbenzene suggests the activation of ethylbenzene in our electrochemical system. (**b**) Linear sweep voltammetry curves of W_2C/N_xC and bare carbon cloth as anodes under standard reaction conditions in the presence of ethylbenzene. The decreased overpotentials by the W_2C/N_xC-based anodes further confirm the role of W_2C/N_xC materials as active components for the activation of ethylbenzene.

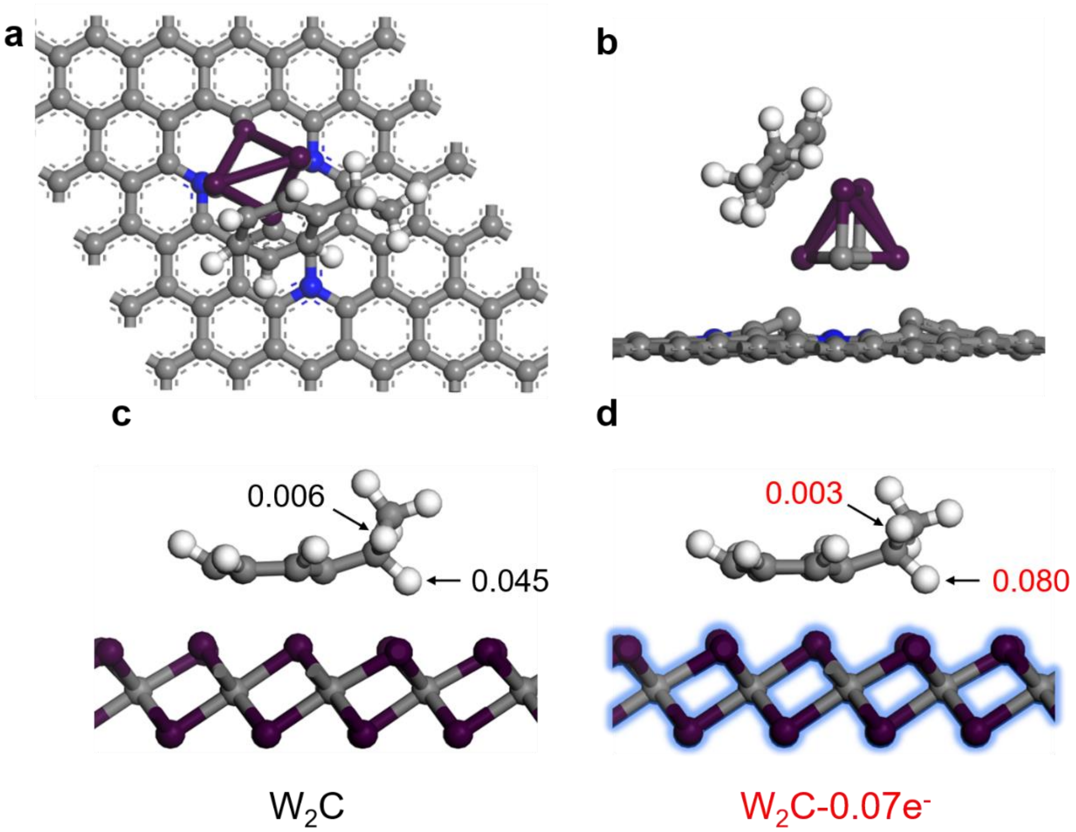

Supplementary Figure 24. The adsorption models of ethylbenzene on W_2C cluster (**a**) top view and (**b**) side view. The ethylbenzene molecule moves closer to the W_2C cluster and far away from the NC support while the benzylic C-H bond is adsorbed around the W atom. The electron numbers from benzylic H atom of ethylbenzene molecule to the surface of W_2C (**c**) and W_2C-0.07e^- (**d**) models. The significant difference in Hirshfeld charge of benzylic H atoms on the electron-deficient W_2C surface directly certifies the polarization role of benzylic C−H bonds of ethylbenzene.

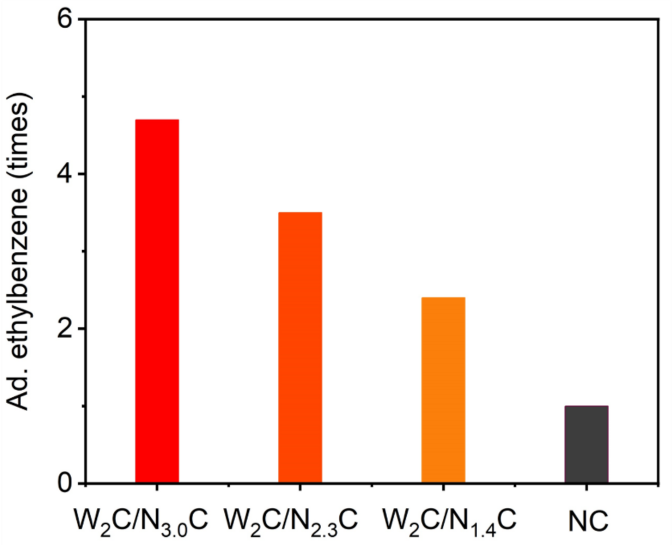

Supplementary Figure 25. The relative adsorption volumes of ethylbenzene absorbed by W_2C/N_xC samples and bare NC support, which are 4.7, 3.5 and 2.4 times for $W_2C/N_{3.0}C$, $W_2C/N_{2.3}C$ and $W_2C/N_{1.4}C$, respectively.

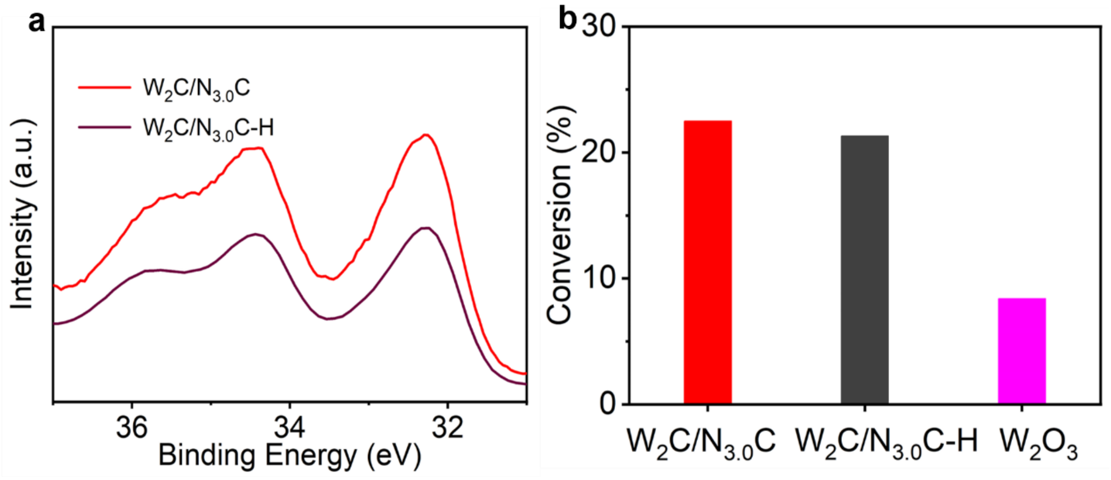

Supplementary Figure 26. The negligible effect of oxygen residues in $W_2C/N_{3.0}C$ on the final activity. W 4*f* XPS spectra (**a**) and conversions (**b**) of $W_2C/N_{3.0}C$, the reduced $W_2C/N_{3.0}C$ sample ($W_2C/N_{3.0}C$-H) and W_2O_3. The $W_2C/N_{3.0}C$ material was reduced by a mixed gas of H_2 (5 %) and Ar (95 %) at 500 °C for 2 h to remove all exposable oxygen active species. Oxygen contents and the activity of $W_2C/N_{3.0}C$ electrode materials remain similar after such a reduction process, demonstrating that the oxygen species, mainly in the form of metal oxides as the minority of W components, is not exposable to reaction environment for possible reactions. Moreover, a poor conversion of ethylbenzene on the control W_2O_3 anode with the same loading of $W_2C/N_{3.0}C$ under standard conditions further exclude the possible role of W_2O_3 as active sites in our electrochemical synthesis system.

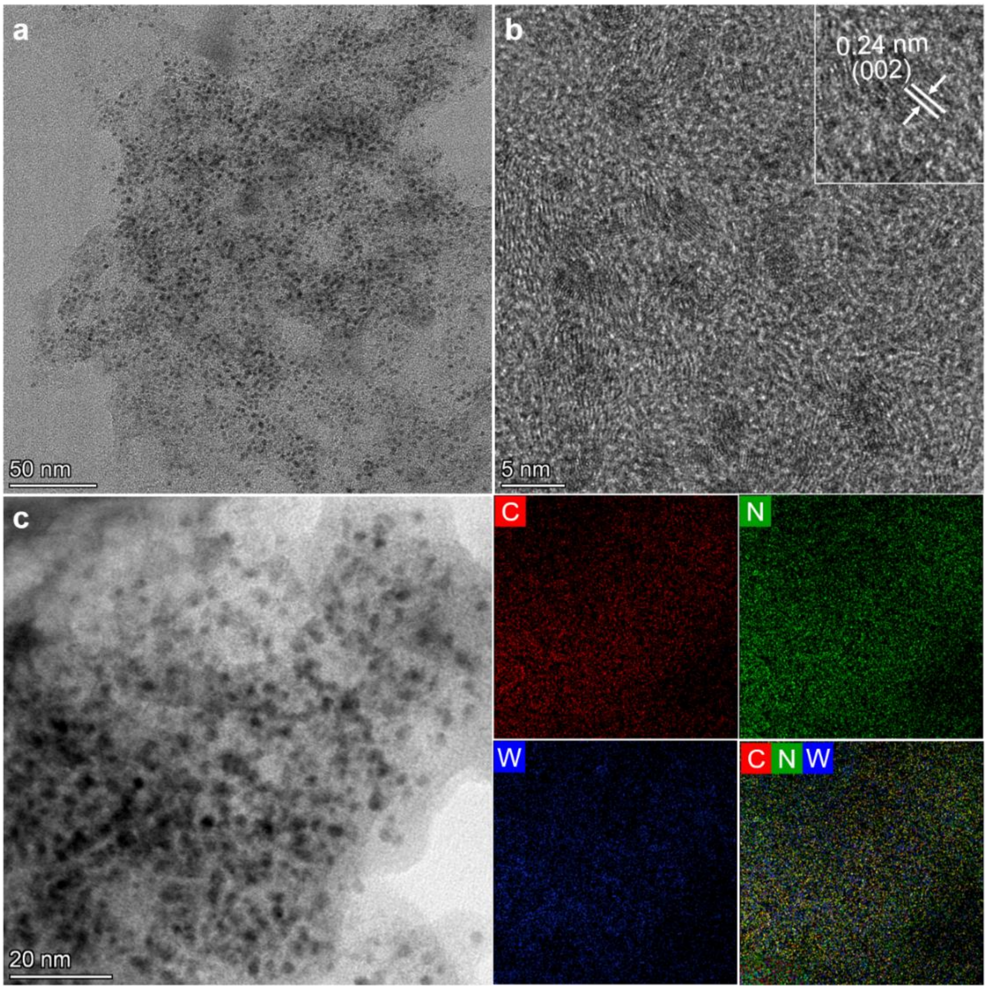

Supplementary Figure 27. TEM (**a**) and HRTEM (**b**) images of $W_2C/N_{3.0}C$-used (recycle 4 times). (**c**) STEM image and EDX elemental mapping of $W_2C/N_{3.0}C$-used. The sizes of W_2C nanocrystals and content of NC support in the $W_2C/N_{3.0}C$ anode were maintained well after long-term use, demonstrating the excellent electrochemical stability of $W_2C/N_{3.0}C$ electrode materials.

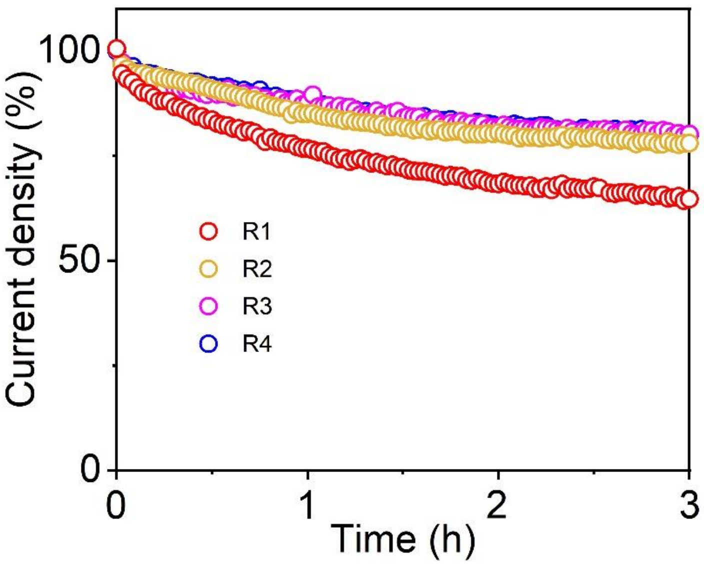

Supplementary Figure 28. Reusability of $W_2C/N_{3.0}C$ anodes for four runs (R1-4).

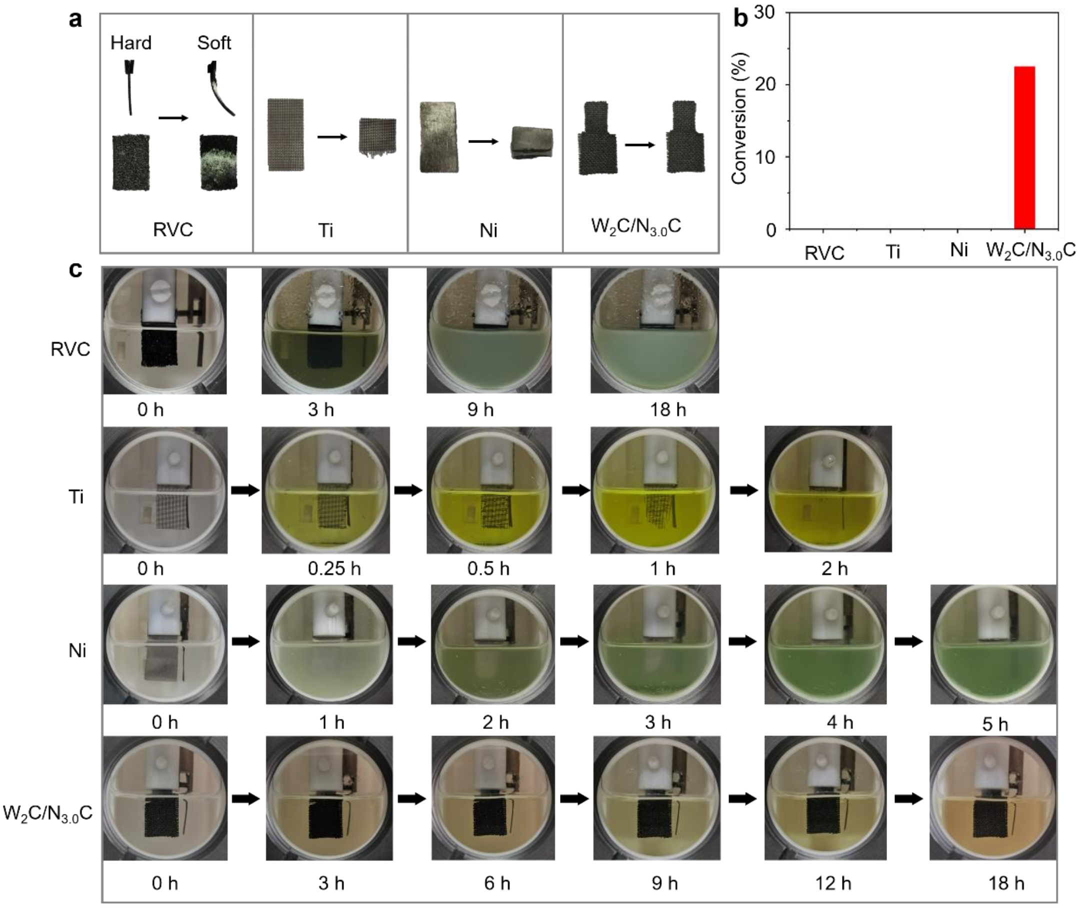

Supplementary Figure 29. The electrochemical stability of $W_2C/N_{3.0}C$, reticulated vitreous carbon (RVC), titanium mesh (Ti) and nickel plate (Ni) anodes. (**a**) The photos of electrodes before and after uses under standard reaction conditions. The Ti and Ni electrodes became decomposed during the reaction. (**b**) Conversions of ethylbenzene on various anodes at 2.0 V for 3 h. No products were detected on RVC, Ni and Ti anodes under standard reaction conditions for 3 h, further highlighting the excellent stability of $W_2C/N_{3.0}C$ anode. (**c**) The photos of RVC, Ti, Ni and $W_2C/N_{3.0}C$ anodes during the reaction with different times.

Supplementary Table 1. Elemental compositions content of W_2C/N_xC samples.

Element / Sample	Atomic ratio (%)		W (wt%) by	W (wt%) by
	C	N	XPS	ICP
$W_2C/N_{3.0}C$	87.2	3.0	50.7	65.8
$W_2C/N_{2.3}C$	88.3	2.3	50.0	62.4
$W_2C/N_{1.4}C$	89.5	1.4	50.9	63.2

Supplementary Table 2. Summary of TOF values of various catalysts for the methoxylation of benzylic C–H bonds.

Catalyst	TOF (h^{-1})	Substrate	Notes	T (°C)	Reference
$W_2C/N_{3.0}C$	18.8	Ethylbenzene	-	rt	This work
CuCl + 2,2′-bioxazoline [a]	0.5	Ethylbenzene	NFSI, $(MeO)_2P(O)H$	40	43
$[Ir(dF(CF_3)ppy)_2(5,5'\text{-}dCF_3bpy)]PF_6$ [b]	12.2	4-ethylanisole	$Cu(TFA)_2(MeCN)$, 427 nm blue LED	rt	44

Reaction conditions: [a] substrate (0.2 mmol), MeOH (1 mmol), CuCl (10 mol%), 2,2′-bioxazoline (10 mol%), *N*-fluorobenzenesulfonimide (NFSI) (2.0 equiv.), $(MeO)_2P(O)H$ (0.5 equiv.), dichloromethane (DCM): hexafluoroisopropanol (HFIP) = 4:1 (1.0 ml) and 16 h under N_2. [b] substrate (0.6 mmol, 1 equiv.), $[Ir(dF(CF_3)ppy)_2(5,5'\text{-}dCF_3bpy)]PF_6$ (1 mol%), K_2HPO_4 (3 equiv), $Cu(TFA)_2(MeCN)$ (1.2 equiv), methanol (2 equiv), MeCN (0.2 M), 6 h.

Supplementary Table 3. TOF values of $W_2C/N_{3.0}C$ heterogeneous catalyst in the alkoxylation of C−H bonds.

Heterogenous catalyst	TOF (h^{-1})	Substrate	Notes	T (°C)	Reference
$W_2C/N_{3.0}C$	18.8	Ethylbenzene	2.0 V versus SCE	rt	This work

Supplementary Table 4. Summary of TOF values of homogeneous catalyst in the functionalization of C−H bonds.

Homogeneous catalyst	Current collector	TOF (h^{-1})	Substrate	Notes	T (°C)	Reference
$Ni(DME)Cl_2$	RVC	0.6	Amides	NaO_2CAd, 1-$AdCO_2H$, 8 mA	130	3
$Co(OAc)_2\cdot 4H_2O$	Carbon cloth	1.2	N-(quinolin-8-yl)thiophene-2-carbox amide	NaOPiv· H_2O, 10 mA	65	4
$Cp^*Rh(OAc)_2$	RVC	2.2	N-(2-pyridyl) aniline	3 mA	65	5
(TAML)Fe	Graphite	0.8	Ethylbenzene	1.25 V versus Ag/AgCl	rt	6
$Pd(OAc)_2$	Graphite	1.2	8-methylquinoline	Benzoquinone, 8 mA	35	7
$PdCl_2$	Pt	3.1	2-phenylpyridine	20 mA	90	8

Supplementary References

1. Latimer, A. A. et al. Understanding trends in C–H bond activation in heterogeneous catalysis. *Nat. Mater.* **16**, 225–229 (2017).
2. Liu, J. et al. Metal-free efficient photocatalyst for stable visible water splitting via a two-electron pathway. *Science* **347,** 970–974 (2015).
3. Zhang, S. K., Struwe, J., Hu, L. & Ackermann, L. Nickela ‐ electrocatalyzed C−H alkoxylation with secondary alcohols: Oxidation ‐ induced reductive elimination at Nickel (III). *Angew. Chem. Int. Ed.* **59,** 3178–3183 (2020).
4. Gao, X., Wang, P., Zeng, L., Tang, S. & Lei, A. Cobalt (II)-catalyzed electrooxidative C–H amination of arenes with alkylamines. *J. Am. Chem. Soc.* **140,** 4195–4199 (2018).
5. Wu, Z. J. et al. Scalable rhodium (III) ‐ catalyzed aryl C−H phosphorylation enabled by anodic oxidation induced reductive elimination. *Angew. Chem. Int. Ed.* **58,** 16770–16774 (2019).
6. Yang, Q.-L. et al. Electrochemistry-enabled Ir-catalyzed vinylic C–H functionalization. *J. Am. Chem. Soc.* **141,** 18970–18976 (2019).
7. Das, A., Nutting, J. E. & Stahl, S. S. Electrochemical C–H oxygenation and alcohol dehydrogenation involving Fe-oxo species using water as the oxygen source. *Chem. Sci.* **10,** 7542–7548 (2019).
8. Kakiuchi, F. et al. Palladium-catalyzed aromatic C-H halogenation with hydrogen halides by means of electrochemical oxidation. *J. Am. Chem. Soc.* **131,** 11310–11311 (2009).
