# Supplementary material for: Electrochemical activation of C–H by electron-deficient W2C nanocrystals for simultaneous alkoxylation and hydrogen evolution
Source: Nat Commun. 2021 Jun 23;12:3882. doi: 10.1038/s41467-021-24203-8 (PMC8222219; doi:10.1038/s41467-021-24203-8)
Supplement: Supplementary file 3 — Description of additional supplementary files [file 41467_2021_24203_MOESM3_ESM.docx]

Description of additional supplementary information file

Title: supplementary movie

Description: phenomenon of hydrogen evolution on the anode.
